# Surface-exposed chaperonin 60 derived from *Propionibacterium freudenreichii* MJ2 inhibits adipogenesis by decreasing the expression of C/EBPα/PPARγ

**DOI:** 10.1038/s41598-023-46436-x

**Published:** 2023-11-07

**Authors:** Mirae An, Young-Hee Lim

**Affiliations:** 1 Department of Healthcare Sciences, Graduate School, Korea University, Seoul, 02841 Republic of Korea; 2 School of Biosystems and Biomedical Sciences, Korea University, Seoul, 02841 Republic of Korea; 3 Department of Laboratory Medicine, Korea University Guro Hospital, Seoul, 08308 Republic of Korea

**Keywords:** Cell biology, Microbiology

## Abstract

Recent studies have shown that the health benefits of probiotics are not limited to those offered by living bacteria. It was reported that both live and killed cells of *Propionibacterium freudenreichii* MJ2 (MJ2) isolated from raw milk showed antiobesity activity in 3T3-L1 cells and high-fat diet-induced obese mice. This study was aimed at identifying the active component(s) responsible for the antiadipogenic activity of MJ2. Cell wall, surface protein, and cytoplasmic fractions of MJ2 were investigated for their inhibitory effects on adipogenesis in 3T3-L1 cells. Adipocytes treated with the surface protein fraction showed significantly lower lipid accumulation. Using the MASCOT algorithm following LC-MS/MS analysis, 131 surface proteins were identified and they were principally classified into three categories (network clusters related to ribosomes, carbon metabolism, and chaperones). Among them, chaperonin 60 (Cpn60) was selected as a potential candidate protein. Cpn60 inhibited lipid accumulation and adipogenesis during the early period of differentiation (days 0–2) and decreased expression of genes related to adipogenesis (*Pparg* and *Cebpa*) and lipogenesis (*Fas* and *Scd1*). The expression of *Gata2/3*, which suppresses adipogenesis, significantly increased in Cpn60-treated cells. Moreover, the nuclear translocation of C/EBPβ was inhibited by Cpn60 treatment. In conclusion, Cpn60, a surface protein in MJ2, shows antiadipogenic activity by reducing the expression of C/EBPβ through the upregulation of *Gata2/3* expression followed by downregulation of *Pparg* and *Cebpa* expression.

Health concerns related to obesity are increasing worldwide because of the correlation between obesity and serious metabolic syndromes, such as type 2 diabetes, nonalcoholic fatty liver diseases, and insulin resistance^1^. Obesity, due to an imbalance in the generation and expenditure of energy, is caused by remodeling of the adipose tissue, which is mainly composed of mature adipocytes and various other cells, such as preadipocytes, mesenchymal stromal or stem cells, immune cells, and vascular epithelial cells^2^. Adipogenesis is the development of adipocytes from mesenchymal stem cells (MSCs). Hypertrophy and hyperplasia of adipocytes result in the development of obesity. Therefore, novel molecules or mechanisms that inhibit adipogenesis are required to prevent or treat obesity^3^. Adipogenesis from multipotent MSCs proceeds through the following stages: MSC is committed to the adipocyte lineage, mitotic clonal expansion (MCE), which replicates DNA and duplicates cells, and terminal differentiation stage in which major transcription factors related to adipogenesis, namely peroxisome proliferator activated receptor γ (PPARγ) and CCAAT/enhancer-binding protein α (C/EBPα) and genes related to lipogenesis, such as fatty acid synthase (FAS) and stearoyl-CoA desaturase (SCD1), are expressed^4^. These processes are regulated by a complex network of transcription factors and signaling pathways^5^. The master adipogenic regulators, PPARγ and C/EBPα, are induced to an epigenomic transition state to lead a transcriptional cascade during the early stage of cellular differentiation^6^.

The health-promoting effects of probiotics are no longer considered to be limited to live microorganisms. Postbiotics, whose consumption is beneficial, include components, such as metabolites (proteins, peptides, lipids, organic acids, and enzymes) produced by probiotics, bacterial components (cell surface proteins, lipoteichoic acids, teichoic acid, peptidoglycan, and polysaccharides), cell-free supernatants, and lysed bacteria^7^. The beneficial properties of postbiotics have led to various clinical applications, such as in the fields of immunomodulation and antitumor interventions, with advantages related to production technologies and safety vis-à-vis live probiotics^8^. Propionate, a short-chain fatty acid produced by *Propionibacterium freudenreichii*, induces apoptosis in human gastric cancer cells^9^. The surface proteins (SP) of *P. freudenreichii* show immunomodulatory effects by stimulating the expression of the anti-inflammatory cytokine, IL-10^10^. SlpB, a surface-layer protein B of *P. freudenreichii*, is involved in the adhesion of probiotic cells to human colon cells, which increases probiotic-host interactions^11^.

*P. freudenreichii* MJ2 (MJ2), isolated from raw milk, has been investigated for its beneficial physiological activities against obesity, osteoporosis, and rheumatoid arthritis^12–14^. However, the key components of MJ2 responsible for these beneficial effects have not been identified. Similarly, administration of live or heat-killed MJ2 reduced weight gain and insulin resistance in high-fat diet-induced obese mice^12^. Based on our previous study, we hypothesized that MJ2 may function as a postbiotic and may have roles beyond those as a probiotic bacterium. In this study, we identified an active component of MJ2 with antiadipogenic activity as a postbiotic and deciphered the mechanism underlying its inhibition of differentiation of 3T3-L1 preadipocytes into adipocytes.

## Results

### Effect of the various cell fractions from *P. freudenreichii* MJ2 on the viability of 3T3-L1 preadipocytes

3T3-L1 cells were treated with each MJ2 cell fraction from 1 × 10^8^ cells/mL of MJ2 or heat-killed *P. freudenreichii* MJ2 (hkMJ2) for 24 h, and cell viability was measured using the 3-[4,5-dimethylthiazol-2-yl]-2,5-diphenyltetrazolium bromide (MTT) assay. There was no significant decrease in the viability of cells treated with the various MJ2 fractions or hkMJ2 (Supplementary Fig. 1).

### Surface proteins from MJ2 contribute to the inhibition of lipid accumulation during the differentiation of 3T3-L1

**Figure 1:**
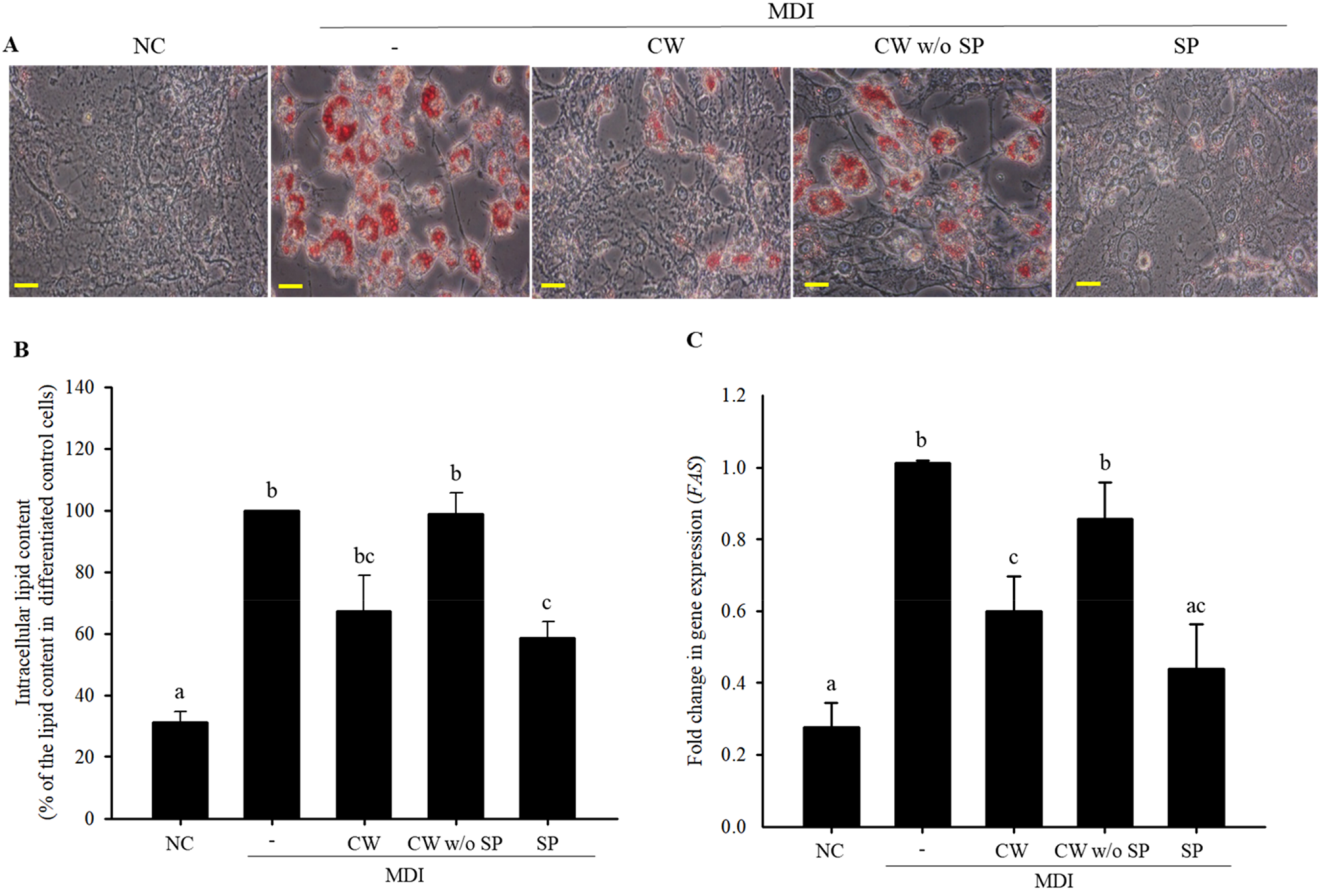
Inhibitory effects of various cell fractions of *Propionibacterium freudenreichii* MJ2 on lipid accumulation in 3T3-L1 cells. 3T3-L1 cells were treated with different cell extracts: cell wall (CW), cell wall without surface proteins (CW w/o SP), and surface proteins (SP) from 1 × 10^8^ *P. freudenreichii* MJ2 cells/mL. (**A**) Lipid droplets in differentiated cells were visualized using Oil Red O staining (× 400 magnification). Scale bars indicate 0.5 mm. (**B**) Lipid accumulation was quantified and compared with that in cells treated only with MDI. (**C**) Fold change in the gene expression of lipogenic transcription factor, *Fas*. The data indicate the mean ± SD of values from three independent experiments. The *p* values were determined using ANOVA and Tukey’s honest significant difference test. Different letters indicate statistically significant differences (*p* < 0.05). NC, negative control; MDI, 1 μM dexamethasone, 0.5 mM IBMX, and 5 μg/mL insulin.

To investigate the effect of each MJ2 cell fraction on lipid accumulation, 3T3-L1 cells were treated with hkMJ2, or cell wall (CW) or cytoplasmic (Cyto) fractions during the induction of cell differentiation. Lipid accumulation decreased in hkMJ2- and CW-treated cells compared with that in differentiated control cells treated only with MDI (3-isobutyl-1-methylxanthine, dexamethasone, and insulin) (Supplementary Fig. 2), which indicates that the active component(s) that decrease lipid accumulation during adipocyte differentiation might be present in the CW. Thus, we measured lipid accumulation in cells treated with CW, CW without surface proteins (CW w/o SP), or SP fractions to specifically identify the active fraction. We found that the SP fraction of CW significantly inhibited lipid accumulation (Fig. 1A,B). In addition, the expression level of the lipogenesis factor, *Fas*, significantly decreased in CW- and SP-treated cells, but not in CW w/o SP-treated cells, compared with that in control cells that were treated only with MDI (Fig. 1C). These results suggest that the candidate active component(s) are present in the SP fraction.

### Characterization of SP isolated from MJ2

**Figure 2:**
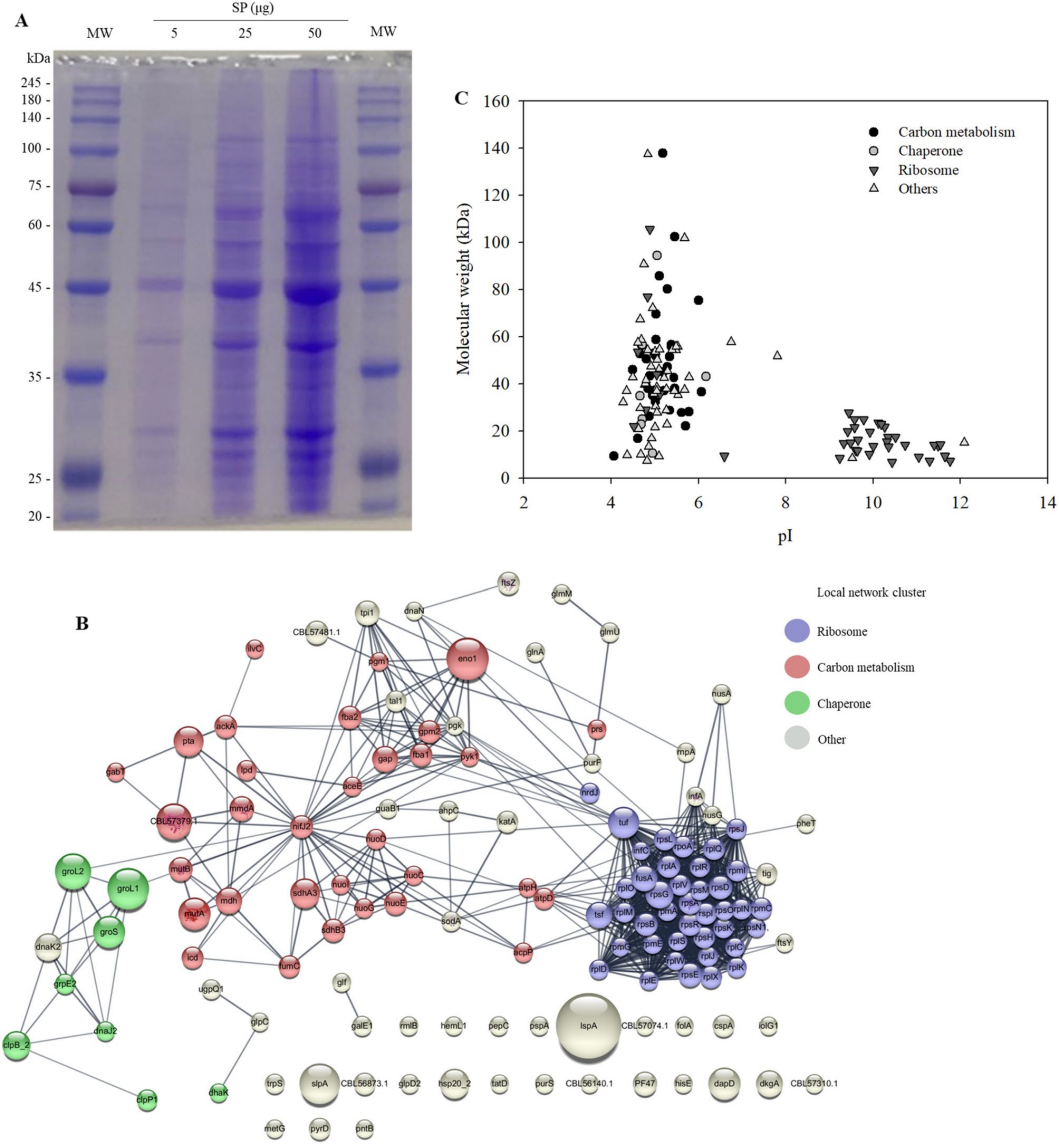
Characterization of surface proteins (SP) isolated from *Propionibacterium freudenreichii* MJ2 using 5 M guanidine hydrochloride. (**A**) SP (5‒50 μg) were separated by electrophoresis on 10% sodium dodecyl sulfate–polyacrylamide gel. The full-length SDS-PAGE image of SP is shown in Supplementary Fig. 3. (**B**) STRING analysis of interaction networks among SP. Proteins are presented as nodes connected by edges. The size of nodes represents the protein score analyzed using the MASCOT algorithm and the color of the node indicates the local network cluster defined using STRING. The interactions of proteins were assessed under a high confidence cutoff (0.7). Purple, red, and green nodes indicate the network clusters related to ribosome, carbon metabolism, and chaperone, respectively. The thickness of edges indicates the confidence level of protein interaction score. (**C**) Molecular weight and isoelectric point (pI) of the identified proteins.

We analyzed the SP fraction using sodium dodecyl sulfate–polyacrylamide gel electrophoresis (SDS-PAGE; Fig. 2A), followed by peptide analysis using nano LC-MS/MS analysis. A total of 131 proteins were identified in the SP fraction of MJ2 (Supplementary Table 1). The molecular weight of these proteins was in the range of 7–138 kDa and the pI was in the range of 4.07–12.09 (Fig. 2B). The results of metabolomic pathway analysis, and Kyoto encyclopedia of genes and genomes (KEGG) classification and gene ontology (GO) analysis of proteins are provided in Supplementary Tables 2 and 3, respectively. The results showed the presence of several characteristic SPs, which were principally classified into three categories (network clusters related to ribosomes, carbon metabolism, and chaperones) (Fig. 2C).

### Chaperonin 60, an MJ2 SP inhibits lipid accumulation in 3T3-L1 cells

**Figure 3:**
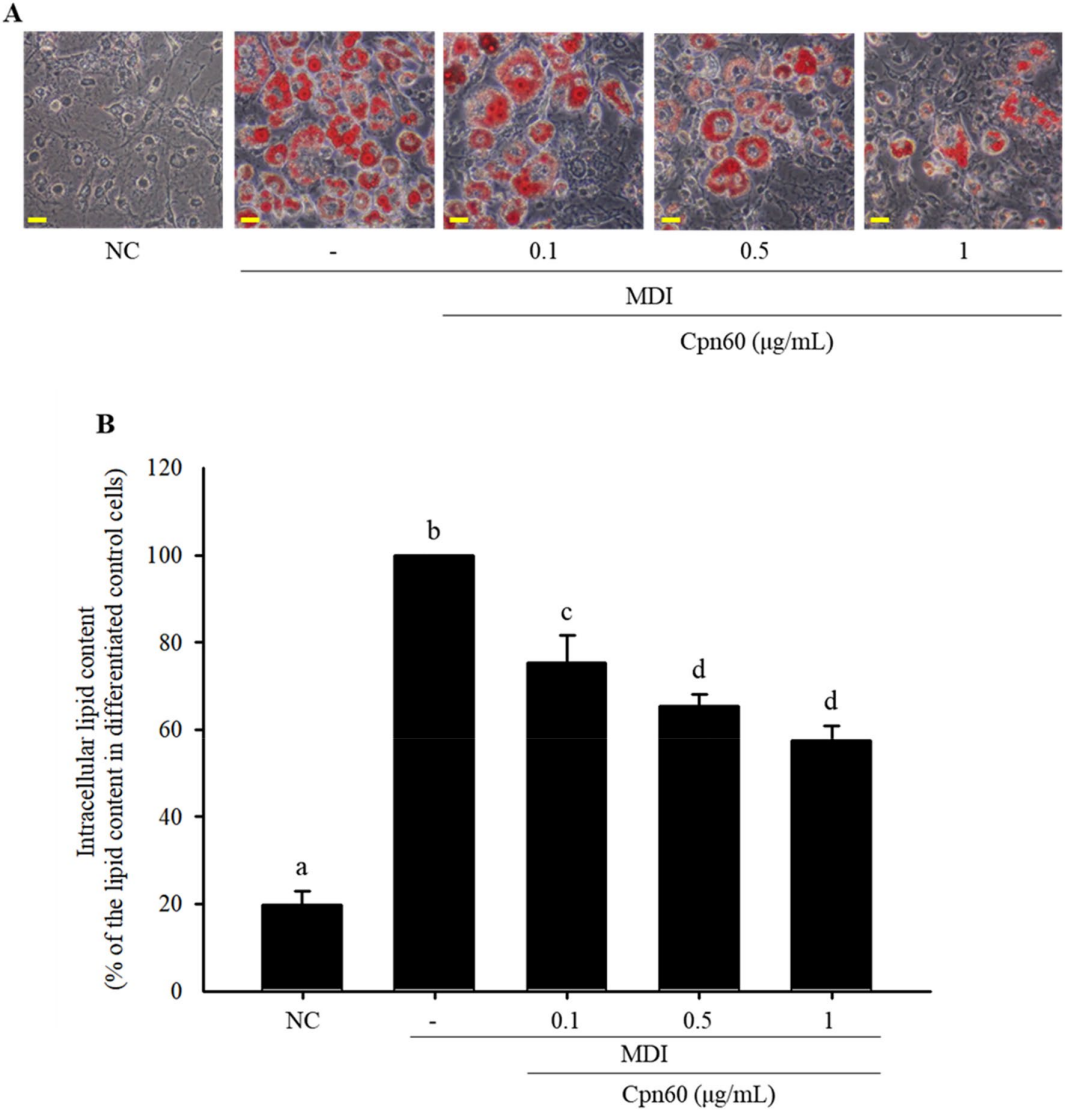
Inhibitory effect of chaperonin 60 (Cpn60) on lipid accumulation in 3T3-L1 adipocytes. 3T3-L1 cells were differentiated using MDI and treated with various concentrations of Cpn60. (**A**) Lipid droplets in differentiated cells were visualized using Oil Red O staining (× 400 magnification). Scale bars indicate 0.5 mm. (**B**) Intracellular lipid content was quantified and compared with that in cells treated only with MDI. The data indicate the mean ± SD of values from three independent experiments. The *p* values were determined using ANOVA and Tukey’s honest significant difference test. Different letters indicate statistically significant differences (*p* < 0.05). NC, negative control; MDI, 1 μM dexamethasone, 0.5 mM IBMX, and 5 μg/mL insulin.

**Figure 4:**
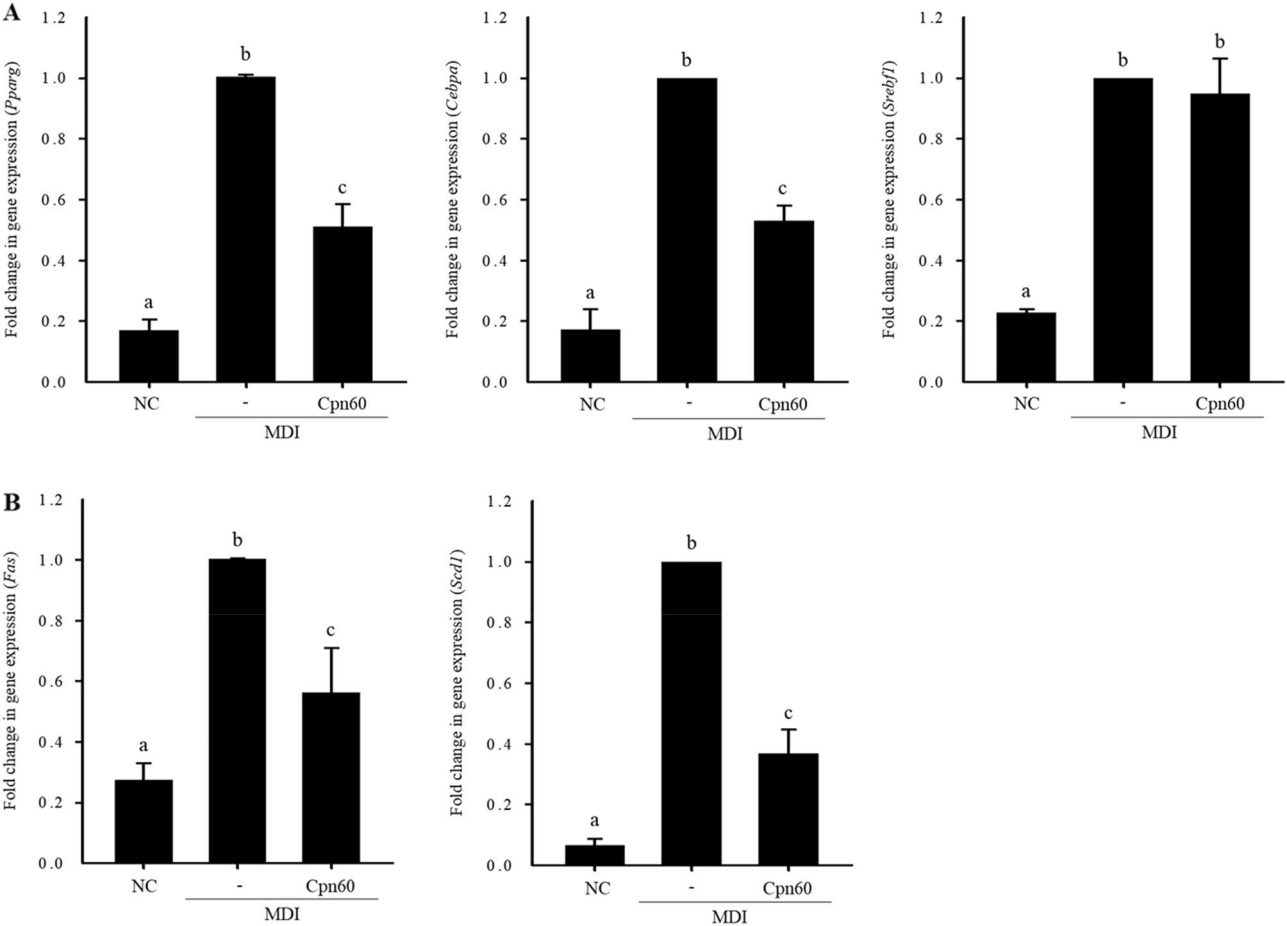
Inhibitory effect of chaperonin 60 (Cpn60) on the expression of genes related to adipogenesis and lipogenesis in 3T3-L1 adipocytes. 3T3-L1 cells were cultured with MDI or MDI + 1 μg/mL Cpn60 and mRNA expression of genes was analyzed using qPCR. Fold change in the expression of adipogenic transcription factor (*Pparg*, *Cebpa*, and *Srebf1*) (**A**) and lipogenic transcription factor (*Fas* and *Scd1*) genes (**B**). The data indicate the mean ± SD of values from three independent experiments. The *p* values were determined using ANOVA and Tukey’s honest significant difference test. Different letters indicate statistically significant differences (*p* < 0.05). *NC* negative control, MDI, 1 μM dexamethasone, 0.5 mM IBMX, and 5 μg/mL insulin.

To select an active candidate for anti-adipogenesis, we fractionated the SP fraction by ion-exchange chromatography using a Q fast column, an anion exchange column, and size exclusive chromatography and investigated the effect of each fraction on lipid accumulation. The fraction strongly bound to Q fast column significantly decreased lipid accumulation, which means that the candidate protein(s) is acidic with relatively low pI (Supplementary Fig. 4). Next, size exclusion chromatography using Superdex 75 was carried out. The results showed that proteins with relatively small molecular weight (less than 20) did not affect lipid accumulation. Thus, we assumed that the active component(s) in SP might be an acidic protein with a molecular weight of more than 35 kDa. Therefore, chaperonin 60 (Cpn60) was selected as a potent active component among the proteins in the SP faction. We synthesized recombinant Cpn60 (Supplementary Fig. 5) and investigated its antiadipogenic effects. Accumulation of lipids in Cpn60-treated 3T3-L1 cells was significantly reduced in a dose-dependent manner compared with that in 3T3-L1 cells treated only with MDI (Fig. 3). The expression of genes related to adipogenesis (*Pparg*, *Cebpa*, and *Srebf1*) and lipogenesis (*Fas* and *Scd1*) was measured to confirm the effect of Cpn60 on the inhibition of adipogenesis. In 3T3-L1 cells treated with 1 μg/mL of Cpn60, the expression of *Pparg*, *Cepba*, *Fas*, and *Scd1* was significantly decreased compared with that in control cells treated only with MDI (Fig. 4). However, the expression of *Srepb1* did not differ between Cpn60-treated 3T3-L1 cells and control cells. These results showed that Cpn60 may be a potential protein component that exerts antiobesity effects by inhibiting adipogenesis.

### Antiadipogenic effect of chaperonin 60 in 3T3-L1 cells during different periods of differentiation

**Figure 5:**
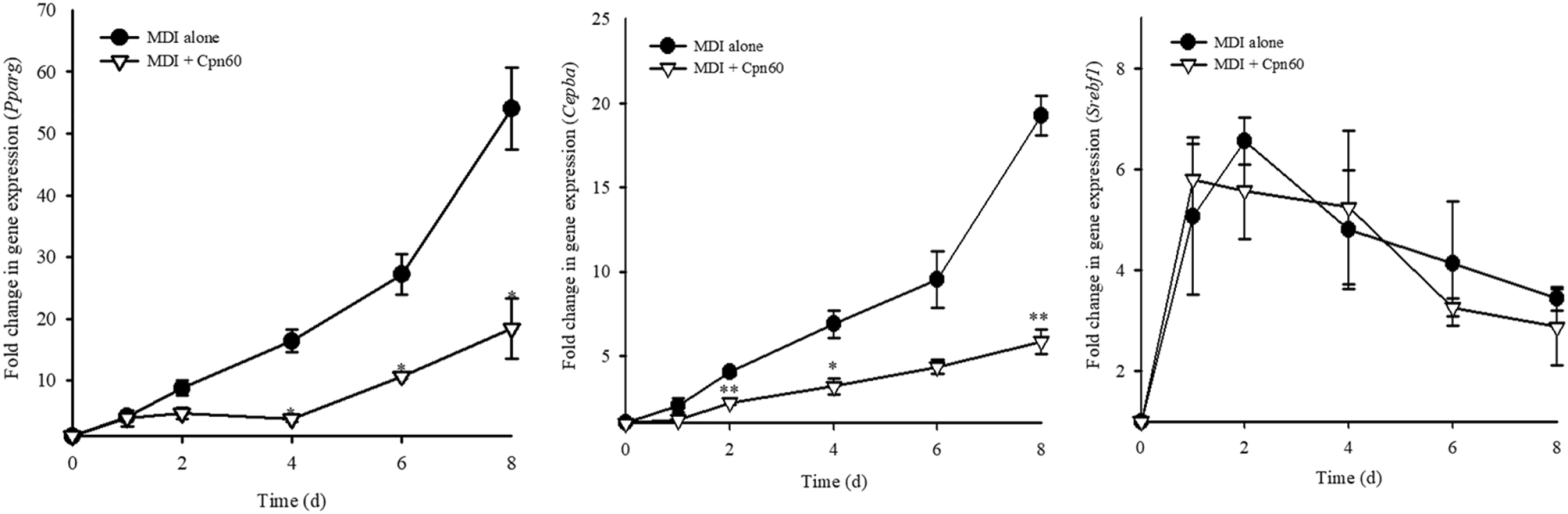
Effect of chaperonin 60 (Cpn60) on the expression of genes related to adipogenesis (*Pparg*, *Cebpa*, and *Srebf1*) in 3T3-L1 adipocytes during different periods of differentiation. The cells were cultured with MDI or MDI + 1 μg/mL Cpn60 for 2, 4, 6, and 8 days and the expression of genes was analyzed using qPCR. The data indicate the mean ± SD of values from three independent experiments. The *p* values were determined using ANOVA and Tukey’s honest significant difference test. Differences were considered significant at *p* < 0.05 (*) and *p* < 0.01 (**). MDI, 1 μM dexamethasone, 0.5 mM IBMX, and 5 μg/mL insulin.

**Figure 6:**
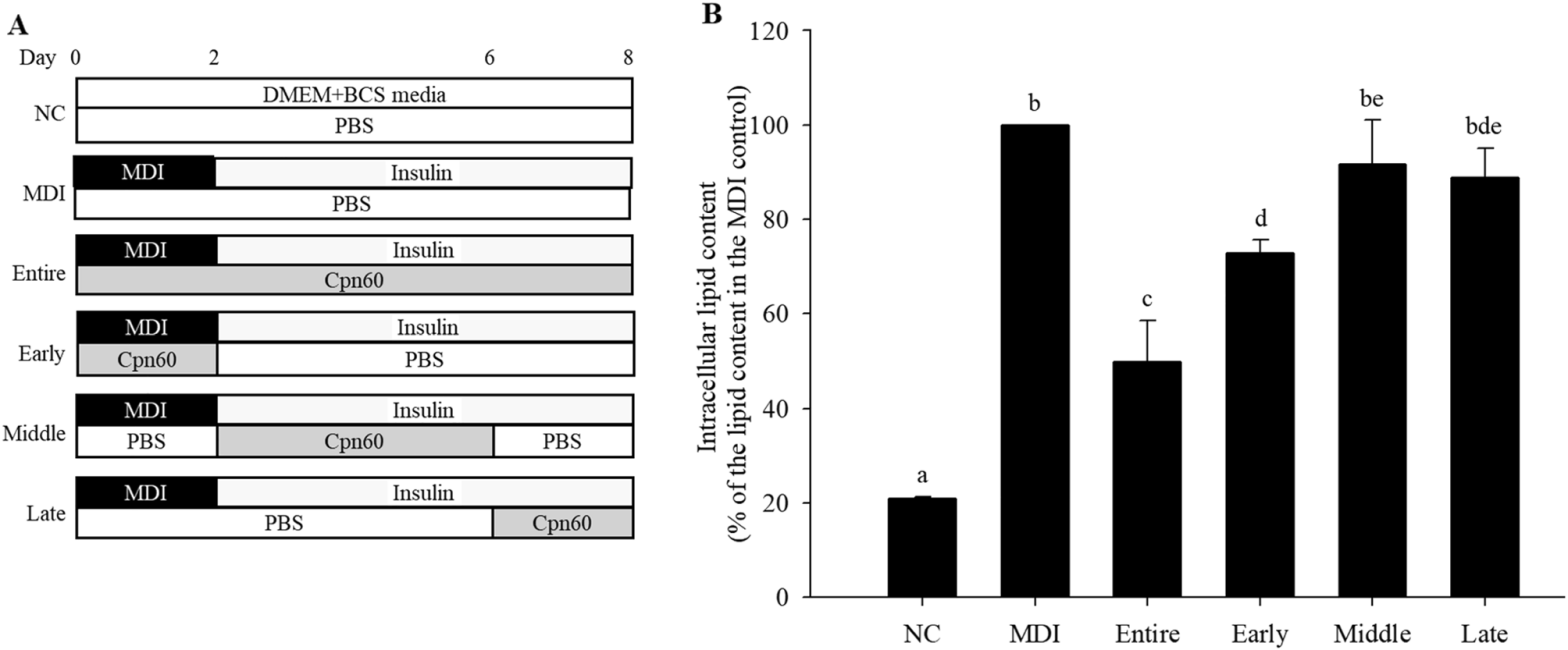
Effect of chaperonin 60 (Cpn60) on lipid accumulation during different periods of treatment of 3T3-L1 cells with Cpn60. 3T3-L1 cells were differentiated by treatment with MDI or MDI + 1 μg/mL Cpn60 for different periods during differentiation (**A**). Lipid accumulation was quantified on day 8 by measuring lipid droplets stained with Oil Red O and comparing them with that in cells treated only with MDI (**B**). The data indicate the mean ± SD of values from three independent experiments. The *p* values were determined using ANOVA and Tukey’s honest significant difference test. Different letters indicate statistically significant differences (*p* < 0.05). NC, negative control; MDI, 1 μM dexamethasone, 0.5 mM IBMX, and 5 μg/mL insulin.

To determine the inhibitory effect of Cpn60 on adipogenesis during the different periods of differentiation, we measured the expression of *Pparg*, *Cebpa*, and *Srebf1* every 2 days during the differentiation period (day 8). The expression of *Pparg* and *Cebpa* in Cpn60-treated cells decreased significantly starting day 4 and day 2, respectively, compared with that in control cells treated only with MDI. In contrast, the expression of *Srebf1* did not differ between Cpn60-treated and control cells during the entire period (Fig. 5). These results are consistent with the gene expression results shown in Fig. 4. Adipogenesis master transcription factors, PPARγ and C/EBPα, act during the early period of cell differentiation, whereas SREBP1 is expressed relatively during the later stages of adipogenesis. We measured lipid accumulation in the different periods of differentiation by treating cells with Cpn60 during the early (day 0‒2), intermediate (day 2‒6), and late (day 6‒8) periods as well as during the entire differentiation period (entire) (Fig. 6A). The accumulation of lipid droplets in Cpn60-traeated cells was significantly decreased during the entire and early periods compared with that in the control cells treated only with MDI (Fig. 6B). The results suggest that Cpn60 inhibited adipogenesis by reducing the activity of the master transcription factors, especially PPARγ and C/EBPα, in early period of adipogenesis.

### Chaperonin 60 elevates the gene expression of GATA-binding factor 2 and 3 (*Gata2* and *Gata3*) and inhibits transcriptional activity of C/EPBβ

**Figure 7:**
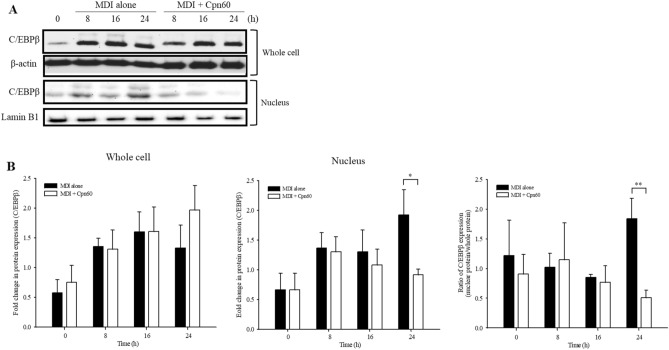
Effects of chaperonin 60 (Cpn60) on the activation of C/EBPβ. 3T3-L1 cells were cultured with MDI or MDI + 1 μg/mL Cpn60 for 0, 8, 16, and 24 h and total and nuclear proteins were extracted. The relative protein levels of C/EBPβ were assessed using western blot analysis (**A**) and quantified densitometrically (**B**). The *p* values were determined using the Student’s *t*-test. Differences were considered significant at *p* < 0.05 (*) and *p* < 0.01 (**). The full-length images of the blots are shown in Supplementary Fig. 7. MDI, 1 μM dexamethasone, 0.5 mM IBMX, and 5 μg/mL insulin.

The expression and activity of PPARγ and C/EBPα, the major regulators of adipogenesis, are regulated through various signaling pathways^2,6,15,16^. We found that the expression of *Pparg* and *Cebpa* was regulated in the early period of adipogenesis upon treatment of 3T3-L1 cells with Cpn60. C/EBPβ is the major transcription factor that initiates adipogenesis induced by MDI and induces the expression of *Pparg* and *Cebpa*. Active C/EBPβ is translocated to the nucleus and induces the transcription of target genes^17^. To investigate the effect of Cpn60 on the nuclear translocation of C/EBPβ, the expression of C/EBPβ was measured during the early period of adipogenesis. The expression of C/EBPβ did not show any significant difference between control and Cpn60-treated cells during the initial 24 h (Fig. 7). However, the level of C/EBPβ in the nucleus was significantly decreased in Cpn60-treated cells at 24 h compared with that in control cells treated only with MDI.

**Figure 8:**
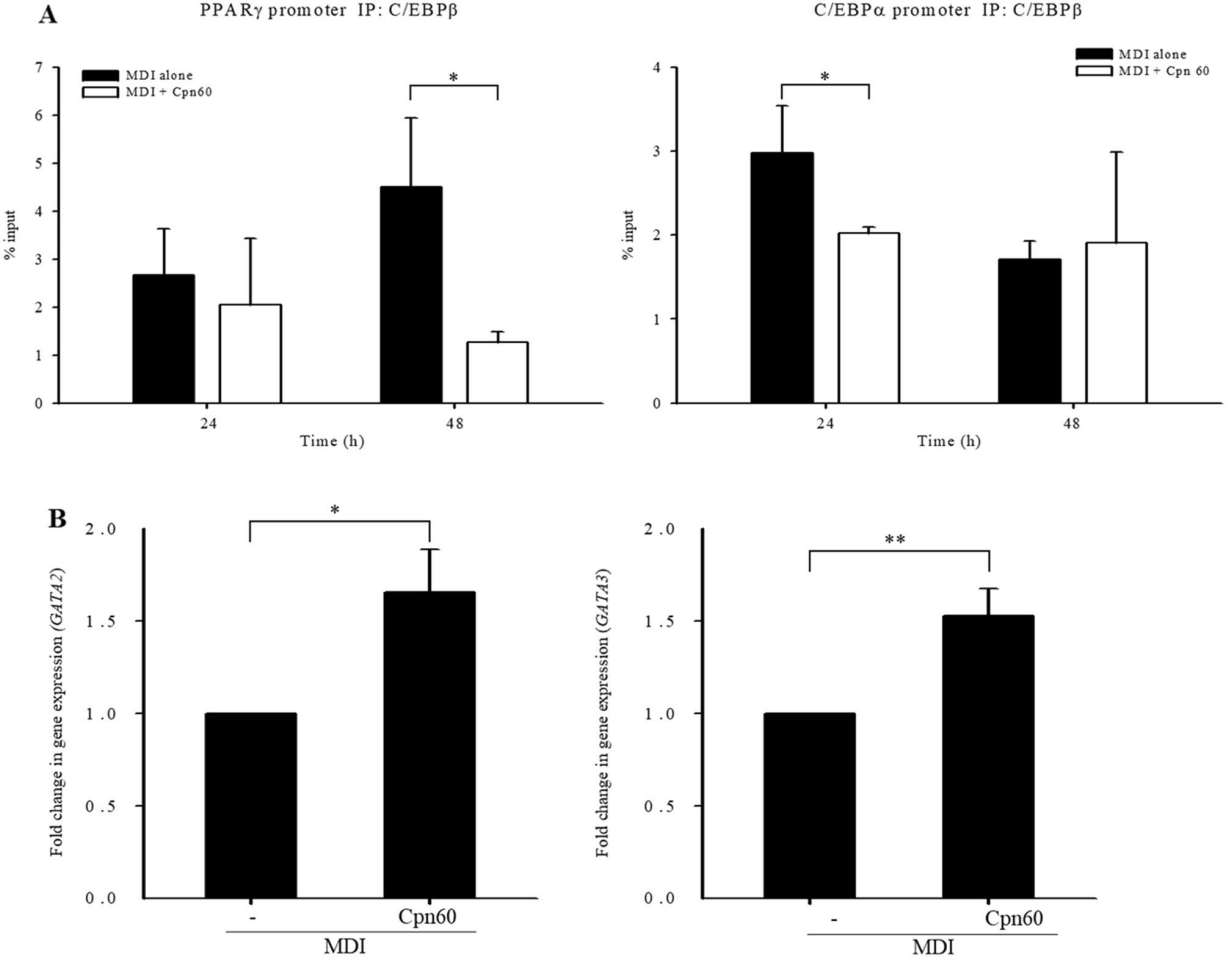
Binding of C/EBPβ on PPARγ or C/EBPα promoter and the expression of *Gata2* and *Gata3* investigated using chromatin immunoprecipitation (ChIP) assay. The cells were cultured with MDI or MDI + 1 μg/mL Cpn60 for 24 and 48 h and the binding of C/EBPβ on PPARγ or C/EBPα promoter was investigated using the ChIP assay (**A**). Fold change in the expression of *Gata2* and *Gata3* was analyzed using qPCR (**B**). The data indicate the mean ± SD of values from three independent experiments. The *p* values were determined using the Student’s *t*-test. Differences were considered significant at *p* < 0.05 (*) and *p* < 0.01 (**). MDI, 1 μM dexamethasone, 0.5 mM IBMX, and 5 μg/mL insulin.

To evaluate the ability of C/EBPβ to bind to the promoter of major factors inducing adipogenesis, *Pparg* and *Cebpa*, we performed chromatin immunoprecipitation (ChIP) using extract of cells treated with MDI or Cpn60 for 24 or 48 h with an anti-C/EBPβ antibody. The percent input bound on the C/EBPα and PPARγ promoters was significantly decreased in cells treated with Cpn60 for 24 and 48 h, respectively (Fig. 8A).

GATA-binding factor (GATA) family is the transcription factor family comprising six members, among which GATA2 and GATA3 suppress adipocyte differentiation by binding to PPARγ, C/EBPα, and C/EBPβ and suppress their activity during adipogenesis^18^. The expression of *GATA2* and *GATA3* was significantly increased in Cpn60-treated cells compared with that in the control cells treated only with MDI (Fig. 8B). Cpn60 treatment resulted in reduced lipid accumulation in 3T3-L1 cells through the upregulation of *Gata2/3*, which inhibits the expression of *Cebpa* and *Pparg* and the translocation of C/EPBβ into the nucleus, the transcription factors that induce adipogenesis in the early stages of the differentiation period.

## Discussion

The use of probiotics, comprising live bacteria, in health supplements and drugs is fraught with several problems in the manufacturing and distribution processes. Consumption of live bacteria is associated with concerns about side effects, such as systemic infections, including bacteremia, harmful metabolic activities stimulating the immune system, and genetic transfer^19^. Postbiotics exhibit various advantages, such as definite chemical structures, selection of safe dosages, long shelf life without denaturation, and bioactivity and health benefits similar to those of live probiotics^20,21^. Cell wall protein fractions extracted from *Lactobacillus paracasei* showed anticancer effects in human colon carcinoma Caco-2 cells by inducing apoptosis^22^. Lipoteichoic acid, located on the cell membrane of *Bifidobacterium animalis* subsp. *lactis* BPL1, reduced obesity biomarkers in *Caenorhabditis elegans* under hyperglycemic conditions via the IGF-1 pathway^23^. A novel protein, HM0539, secreted by *Lactobacillus rhamnosus* GG, protected the intestinal barrier by reinforcing mucin expression in Caco-2 cells and in neonatal rats infected with *E. coli*^24^. In this study, we found that the surface proteins of MJ2 inhibited lipid accumulation in 3T3-L1 cells, an effect similar to that seen for heat-killed intact MJ2, which indicates the potential of the surface proteins of MJ2 for use as postbiotics.

Moonlighting proteins are proteins produced from a single gene sequence that have more than one function in a cell or organism by sharing genes. Cpn60 is generally localized in the cytoplasm and plays a role in protein folding in many bacteria^25^. However, Cpn60 is a moonlighting protein that performs several other physiologically important biophysical and biochemical functions besides its main functions when localized to other subcellular locations. It is often located on the surface of bacteria^26^. In *Lactococcus lactis*, Cpn60, located on the cell surface, plays a role in binding to invertase from *Saccharomyces cerevisiae*^27^. Cpn60 of *L. johnsonii* La1, located on the cell surface, stimulates interleukin-8 through a CD14-dependent mechanism by binding to mucins and epithelial cells of the host^28^. In the present study, the surface proteins of MJ2 were sorted into four groups—chaperones, proteins involved in carbon metabolism, ribosomal proteins, and usually surface-located proteins. The chaperones and proteins involved in carbon metabolism identified in this study are known as moonlighting proteins that are highly conserved across phyla. Surface proteins isolated from MJ2 are generally located in parts other than the surface and may have functions different from their known functions. Identification of the active components of probiotics and prebiotics and understanding of the mechanisms underlying their beneficial effects are important to obtain maximum benefits with minimal side effects. Various cell fractions isolated from MJ2 were separated and surface fraction showed an inhibitory effect on lipid accumulation. Among the surface proteins, Cpn60, a well-known moonlighting protein, was selected as a potent active component based on its acidity, molecular weight, and moonlighting properties, however other proteins that satisfied the conditions could also be considered active components. Cpn60 was identified as a potentially active antiadipogenic SP of MJ2.

Heat shock protein 60 or chaperonin 60 (HSP60 or Cpn60) is highly conserved among species from bacteria to mammals, however, the sequences of *Hsp60* do not show greater similarity between eukaryotic cells and Gram-positive bacteria like *P. freudenreichii*^29^. Mitochondrial HSP60 modulates energy metabolism in adipose tissue and reduced HSP60 level induces mild mitochondrial dysfunction that causes insufficient mitochondrial energy production resulted in the protection of diet-induced obesity in a high fat diet-induced obese male mice^30^. In this study, the addition of Cpn60 inhibited adipogenesis by regulating expression of adipogenesis-related transcription factors and we do not have direct evidences that the addition of Cpn60 decreases mitochondrial energy production, which induces antiobesity in a high fat-diet induced obese mice. Further studies are required to investigate whether Cpn60 isolated from the SP of *P. freudenreichii* MJ2 shows the antiobesity effect by the modulation of energy metabolism similar with HSP60 from C57BL/6N mice.

In the intestine, proteins regardless of origin usually break down into small peptides and amino acids by mainly microbial proteases and are absorbed cross the intestinal tract. In case of intact proteins, they bind to receptors on the surface of the membrane of intestinal epithelial cells and are translocated into the cytoplasm by endocytic mechanism^31^. In addition, lysosome-rich enterocytes, specialized intestinal cells, internalize proteins via receptor-mediated endocytosis followed by uptake proteins into the intestinal tract by endocytic machinery^32^. Although it is not clear the mechanism of absorption of probiotic-derived secreted proteins in the intestinal tract, Cpn60 from a probiotic bacterium might be absorbed by endocytic mechanism. In this study, we found the inhibitory effects of Cpn60 on adipogenesis in vitro. Thus, further studies need to confirm the effects of Cpn60 on anti-adipogenesis in vivo study and investigate its absorption mechanism in the intestine.

Preadipocytes, including 3T3-L1 preadipocytes, are differentiated into adipocytes through MCE, which is an essential step in differentiation during the early stage of adipogenesis and is followed by induction of the expression of key regulators of adipogenesis, such as PPARγ and C/EBPα. Differentiated adipocytes produce lipid droplets in the cytosol and express mature adipocyte markers, such as adipokines, adipose triglyceride lipase, and perilipin^33^. C/EBPs, comprised of six members, are key factors that regulate cellular differentiation. Among them, C/EBPβ is expressed immediately after the induction of adipogenesis by an adipogenic cocktail (MDI). The expressed C/EBPβ gains DNA binding ability upon phosphorylation by a mitogen-activated protein kinase (MAPK) and then induces expression of transcription factors, PPARγ and C/EBPα, which are critical factors in adipogenesis. Thus, interrupting the processes that occur in the early stages of adipogenesis contributes to the prevention or treatment of obesity. In this study, the translocation of C/EBPβ in preadipocytes was inhibited by Cpn60 treatment, which subsequently decreased the expression of *Pparg* and C/EBPβ, demonstrating the potential of Cpn60 as a therapeutic molecule for obesity.

GATA2/3 forms a complex with C/EBPα and C/EBPβ, which plays a critical role in the ability of GATA to suppress adipocyte differentiation^18^. C/EBPβ is the major transcription factor that initiates adipogenesis induced by MDI and induces the expression of *Pparg* and *Cebpa*. Cpn60 inhibits the activation of C/EBPβ in the nucleus by upregulating the expression of GATA2/3 and subsequently downregulating the expression of PPARγ and C/EBPα, which eventually inhibits adipogenesis and decreases lipid accumulation in 3T3-L1 cells. However, in our further study, it should be investigated whether inhibition of GATA2/3 restores the Cpn60-induced reduction of the DNA binding capacity of C/EBPβ and *Pparg* and *Cebpa* expression. In addition, further studies need to investigate whether inhibition of Cpn60 decreases its anti-adipogenesis activity.

In conclusion, Cpn60 reduced lipid accumulation in 3T3-L1 cells by upregulating the expression of *GATA2/3*, which might hinder the translocation of C/EPBβ and result in the inhibition of the expression of *Cebpa* and *Pparg*, the transcription factors necessary for the induction of adipogenesis in the early stages of differentiation.

## Materials and methods

### Materials

Dulbecco’s modified Eagle’s medium (DMEM), bovine calf serum (BCS), penicillin/streptomycin (P/S), and trypsin-EDTA used for cultivation of cells were purchased from HyClone (Logan, UT, USA). DNaseI, RNase, 3-isobutyl-1-methylxanthine (IBMX), dexamethasone, and insulin were obtained from Sigma-Aldrich (St. Louis, MO, USA). MTT was purchased from Amresco (Solon, OH, USA).

### Bacterial strains and culture conditions

*P. freudenreichii* MJ2 strain (KCCM12272P) (MJ2)^14^ was cultured in Reinforced clostridial medium (RCM) (Oxoid, Hampshire, United Kingdom) at 30 °C under anaerobic conditions for 48 h until the stationary phase was reached. Thereafter, the cells were collected by centrifugation at 8000×*g* for 10 min at 4 °C and then washed twice with 20 mM Tris-HCl (pH 8). Washed cells were used to obtain cell fractions or were heated at 100 °C for 30 min to get hkMJ2. HkMJ2 showed no growth.

### Preparation of bacterial cell fractions

The CW and Cyto fractions of MJ2 cells were extracted as described previously^34–38^. In brief, MJ2 cells were washed, resuspended in 1/10 volume of 20 mM Tris-HCl (pH 8), and incubated at − 80 °C for 2 h. The frozen cell solution was subjected to freeze–thaw cycles to weaken the cell wall. The cells were harvested by centrifugation at 8000×*g* for 10 min at 4 °C and the cell pellet was resuspended in 20 mM Tris-HCl (pH 8). The cells were disrupted by bead beating with sterile glass beads (diameter, 100 μm) and vortexing for 1 min for 15 cycles, and were left on ice for 1 min. The lysate was centrifuged at 1000×*g* for 10 min at 4 °C, and the supernatant, thus obtained, was centrifuged at 16,000×*g* for 10 min at 4 °C to remove unbroken cells. The supernatant was further centrifuged at 40,000×*g* for 10 min at 4 °C. The pellet containing the crude extract of the CW was treated with 50 μg/mL DNaseI and 100 μg/mL RNase A for 4 h at 37 °C and then centrifuged at 40,000×*g* for 10 min at 4 °C. The pellet containing CW was collected and the supernatant was ultracentrifuged at 110,000×*g* for 1 h at 4 °C using an ultracentrifuge (Optima L-100 K, Beckman Coulter, Brea, CA, USA) to fractionate the pellet containing the crude extract of the membrane and the supernatant containing the crude extract of cytoplasm (Cyto). The cell fractionation scheme is shown in Supplementary Fig. 6.

### Preparation of SP from MJ2

The extraction of SP from MJ2 was carried out using two different methods as described previously^10,39^. In brief, MJ2 cells were incubated at 30 °C under anaerobic conditions for 48 h and subsequently collected by centrifugation at 8000×*g* for 10 min at 4 °C. The collected cells were resuspended in 5 M GuHCl (DAEJUNG, Seoul, Korea) to a final OD_600_ of 20 and incubated for 15 min at room temperature with gentle shaking. The suspension was centrifuged at 40,000×*g* for 10 min at 4 °C to collect SP. The supernatant was dialyzed against 20 mM Tris–HCl buffer using a dialysis cassette (MWCO 10 K, Slide-A-Lyzer™, Thermo Fisher Scientific, Waltham, MA, USA) for 24 h at 4 °C. The CW fraction without SP (CW w/o SP) was washed twice with 20 mM Tris-HCl and extracted as described above (Supplementary Fig. 6).

### 3T3-L1 cell culture and adipocyte differentiation

3T3-L1 preadipocytes were purchased from the American Type Culture Collection (ATCC CL-173). The cells were cultured at 37 °C in a humidified 5% CO_2_ environment in DMEM with 10% BCS and 1% P/S. The culture medium was changed every two days. For two days, the cells were maintained at 100% confluence. Thereafter, the culture medium was replaced with the MDI medium (DMEM containing 10% FBS, 1% P/S, and MDI) for inducing adipocyte differentiation. The day on which the culture medium was replaced with the MDI medium was considered day 0. On day 2, the medium was replaced with insulin medium (DMEM containing 5 μg/mL insulin), which was refreshed every two days until day 8.

### Assessment of cell viability

The cell viability was measured by MTT assay to determine the effect of various MJ2 cell fractions on the viability 3T3-L1 preadipocytes. We assessed the same concentration of cell extract from 1 × 10^8^ hkMJ2 cells/mL, which was previously shown to inhibit lipid accumulation in 3T3-L1 cells without cytotoxicity^12^. 3T3-L1 preadipocytes (5 × 10^4^ cells/mL) were seeded in a 96-well plate containing DMEM supplemented with 10% BCS and cultured at 37 °C in a humidified 5% CO_2_ atmosphere for 24 h. The medium was then replaced with DMEM without 10% BCS and treated with various crude cell fractions prepared from 1 × 10^8^ MJ2 cells/mL (CW, Cyto, and SP) or from 1 × 10^8^ hkMJ2 cells/mL. After incubation for 24 h, the medium was discarded and MTT reagent (0.125 mg/mL) was added into the wells. The plate was incubated at 37 °C for 1 h in the dark. The supernatant was completely removed, and 200 μL of dimethyl sulfoxide was added to each well. After 30 min, absorbance was measured at 540 nm using a microplate reader (SpectraMax 340PC, Molecular Devices, Sunnyvale, CA, USA). Relative cell viability (%) compared to that of the negative control was calculated.

### Oil red O staining

Oil Red O staining was performed on day 8 when preadipocytes were differentiated into adipocytes more than 80%^40^. Briefly, cells were washed with PBS twice and fixed with 10% formalin for 1 h at room temperature. The fixed cells were washed twice with deionized water, incubated with isopropanol (60%) for 5 min, and soaked in Oil Red O working solution for 30 min at room temperature. After staining, the cells were washed four times with deionized water and allowed to dry completely. Isopropanol (100%) was added to each well to extract the stain; the solution from each well was transferred to a new microplate, and its absorbance was measured at 500 nm for quantification of the Oil Red O stain. The relative lipid accumulation (%) in cells subjected to each treatment was calculated by comparing with the absorbance of the cells treated only with MDI (differentiation control).

### Quantitative real-time polymerase chain reaction (qPCR)

Total RNA was extracted with TRIzol reagent (Thermo Fisher Scientific) following the manufacturer’s instructions. The concentration of RNA was measured using a NanoDrop spectrophotometer (ND-1000 spectrophotometer, Thermo Fisher Scientific). After quantification, RNA was reverse transcribed into cDNA using a RevertAid First Stand cDNA Synthesis Kit (Thermo Fisher Scientific). cDNA was amplified using qPCR with a KAPA SYBR FAST qPCR Kit (KAPA Biosystems, Woburn, MA, USA) on a QuantStudio 6 Flex system (Life Technologies, Carlsbad, CA, USA). The primers used in this study were purchased from Bioneer (Daejeon, Korea). The sequences of the primers are *Pparg* (Forward 5′-AGA CAT CAG CGC CTA CAT CG-3′, Reverse 5′-GCT CCC GGG TAG TCA AAG TC-3′), *Cebpa* (Forward 5′- TGG ACA AGA ACA GCA ACG AG-3′, Reverse 5′-TCA CTG GTC AAC TCC AGC AC-3′), *Srebf1* (Forward 5′-AAC CAG AAG CTC AAG CAG GA-3′, Reverse 5′-TTT CAT GCC CTC CAT AGA CA-3′), *Scd1* (Forward 5′-TGA CTA TCA TCA TGC CGG CC-3′, Reverse 5′-CTT TGA CAG CCG GGT GTT TG-3′), *Gata2* (Forward 5′-CGA CCA CAC TTG TTG CAC AG-3′, Reverse 5′-GGG TAA ACA GAC AGA GGC CC-3′), *Gata3* (Forward 5′-GCT ACG GTG CAG AGG TAT CC-3′, Reverse 5′-GAG GGT AAA CGG ACA GAG GC-3′), and *β-actin* (Forward 5′-GAC ATG GAG AAG ATC TGG CA-3′, Reverse 5′-GGT CTT TAC GGA TGT CAA CG-3′). The reaction was preheated to 95 °C, and then subjected to 40 cycles of 95 °C for 15 s, 60 °C for 15 s, and 72 °C for 30 s. β-actin was used as the reference gene. The normalized mRNA expression level was indicated as 2^−ΔΔCt^ (the expression level of β-actin control was set to 1)^41^.

### LC-MS/MS for peptides analysis

Nano-LC-MS/MS analysis was performed using an Easy n-LC system (Thermo Fisher Scientific) and an LTQ Orbitrap XL mass spectrometer (Thermo Fisher Scientific) equipped with a nano-electrospray source. Samples were separated on a C18 nanopore column (150 mm × 0.1 mm, 3 μm pore size; Agilent, Santa Clara, CA, USA). Mobile phase A for LC separation comprised of 0.1% formic acid and 3% acetonitrile in deionized water, and mobile phase B was 0.1% formic acid in acetonitrile. The linear solvent gradient profile used was as follows: 0% mobile phase B for 40 min; 40–60% mobile phase B for 4 min; 95% mobile phase B for 4 min; 100% mobile phase A for 6 min. The solvent flow rate was maintained at 1.5 μL/min. Mass spectra were acquired using data-dependent acquisition with a full mass scan (350–1200 m/z), followed by 10 MS/MS scans. For MS1 full scans, the Orbitrap resolution was 15,000, and the automatic gain control (AGC) was 2 × 10^5^. For MS/MS in the LTQ, AGC was 1 × 10^4^. The MASCOT algorithm (Matrix Science, Boston, MA, USA) was used to identify peptide sequences present in the UniProt protein sequence database. The database search criteria were as follows: taxonomy: *Propionibacterium freudenreichii*; fixed modification: carbamidomethylated at cysteine residues; variable modification: oxidized at methionine residues; maximum allowed missed cleavage: 2; MS tolerance: 10 ppm; MS/MS tolerance: 0.8 Da. The peptides were filtered using a significance threshold of *p* < 0.05. The 131 identified proteins in the SP fraction were analyzed using the STRING database version 11, which indicates networks of interactions among proteins. The interactions of proteins were assessed under a high-confidence cutoff (0.7), which indicated the minimum required interaction score. Protein networks were visualized using Cytoscape version 3.7.1.

### Proteins expression and purification

Based on the results of the inhibition of lipid accumulation in 3T3L1 cells treated with the fractions obtained from ion exchange and size exclusive chromatography (Supplementary Fig. 4), Cpn60 was selected as the most likely active substance inhibiting lipid accumulation. To investigate whether Cpn60 has antiobesity properties, recombinant Cpn60 was generated by cloning the MJ2 Cpn60 into the pBT7-N-His plasmid vector (Bioneer) and transforming *Escherichia coli* cells with the vector construct. The transformed *E. coli* was grown at 37 °C in LB broth. Recombinant Cpn60 was purified using an Automated Protein Production System (ExiProgen, Bioneer) and its purity was confirmed by electrophoresing on a 10% SDS-polyacrylamide gel (Supplementary Fig. 5).

### Western blot analysis

Whole protein was extracted from 5 × 10^6^ 3T3-L1 cells using the PRO-PREP™ protein extraction solution (iNtRON Biotechnology, Seongnam, Korea) according to the manufacturer’s instructions. Nuclear and cytoplasmic proteins were extracted using NE-PER Nuclear and Cytoplasmic Extraction Reagents (Thermo Fisher Scientific), according to the manufacturer’s instructions. Protein concentration was determined using Bradford assay with bovine serum albumin as a standard. Each protein sample (10 μg) was separated by electrophoresis on a 10% SDS-polyacrylamide gel. The proteins were transferred onto a polyvinylidene difluoride membrane (0.45 μm, Millipore, Bedford, MA, USA); the membrane was blocked with 6% skimmed milk in Tris-buffered saline with 0.05% Tween 20 (TBST) and incubated with primary antibodies overnight at 4 °C. β-actin antibody (1:10,000 dilution, GTX109639, GeneTex, Irvine, CA, USA) and lamin B1 antibody (1:1000 dilution, GTX103292, GeneTex) were used as endogenous controls for whole and nuclear proteins, respectively. Anti-C/EBPβ (1:1000 dilution, sc-7962; SANTA CRUZ Biotechnology, Dallas, TX, USA) was used as the primary antibody. Membranes were washed with TBST and incubated with secondary antibodies for 1 h at room temperature. Goat anti-rabbit IgG horseradish peroxidase (HRP)-conjugated antibody (1:2000 dilution, GTX213110-01, GeneTex) was used as the secondary antibody for anti-β-actin and anti-lamin B1. A goat anti-mouse IgG antibody (HRP) (1:2000 dilution, GTX213111-01, GeneTex) was used as a secondary antibody for anti-C/EBPβ. The membranes were washed with TBST and the bands were detected with a SuperSignal West Femto Maximum Sensitivity Substrate Kit (Thermo Fisher Scientific) using a FluorChem E system (ProteinSimple, San Jose, CA, USA). Densitometric analysis was performed using the ImageJ software (Softomic, Barcelona, Spain).

### ChIP assay

ChIP assay was performed using a ChIP assay kit (Sigma-Aldrich), according to the manufacturer’s instructions. Briefly, 3T3-L1 preadipocytes were differentiated into adipocytes and treated with 1 μg/mL of Cpn60 for 24 or 48 h. The cells were crosslinked using 1% formaldehyde for 10 min at room temperature, and then formaldehyde was quenched with 2.5 M glycine. The crosslinked cells were washed with ice-cold PBS twice and then harvested by centrifugation at 800×*g* at 4 °C for 5 min. The cell pellet was resuspended in a cell lysis buffer and incubated for 15 min on ice. The lysed cells were centrifuged and the pellet was resuspended in the nuclear lysis buffer. The cell lysate was reacted with 1 unit of micrococcal nuclease (Sigma-Aldrich) with 0.1 M CaCl_2_ at 28 °C to fragment DNA (0.5‒1 kb) and then centrifuged at 10,000×*g* for 10 min at 4 °C to remove insoluble materials. The lysates were diluted with ChIP dilution buffer and 2% of the diluted lysates were removed as input and stored at − 20 °C until immunoprecipitation. The chromatin-protein complexes were immunoprecipitated with 5 μg of anti-C/EBPβ antibody (H-7) (sc-7962, SANTA CRUZ Biotechnology) and 20 μL of protein G magnetic beads at 4 °C for overnight. After immunoprecipitation, protein G-antibody complexes were washed with low-salt immune complex wash buffer, high-salt immune complex wash buffer, LiCl buffer, and TE buffer for 3 min each, and then the protein G–antibody complexes and input were reversed crosslinked by incubating with ChIP elution buffer and proteinase K at 62 °C for 2 h. After reverse crosslinking, DNA was purified using spin-column extraction. Purified DNA was amplified using qPCR on a 7500 Fast Real-Time PCR System (Applied Biosystems). The sequences of primer synthesized by Bioneer: *PPARγ* promoter (Forward 5′-TTC AGA TGT GTG ATT AGG AG-3′, Reverse 5′-AGA CTT GGT ACA TTA CAA GG-3′) and *C/EPBα* promoter (Forward 5′-TCC CTA GTG TTG GCT GGA AG-3′, Reverse 5′-CAG TAG GAT GGT GCC TGC TG-3′). The resulting Ct values from the ChIP analysis were normalized using the percent input method (%input = 100 × 2^Adjusted input—Ct (IP)^).

### Statistical analysis

Statistical Package for the Social Science (SPSS) analysis program (ver. 25) was used for all statistical analyses. The statistical analysis was performed using one-way analysis of variance (ANOVA) and Tukey’s honest significant difference (HSD) method as a post hoc test to confirm significant differences between the groups in case the experiments involved various groups. Single comparisons were performed to evaluate the significance between the negative control and treatment groups using the Student’s *t*-test. Data are presented as mean ± standard deviation (SD). Statistical significance was set at *p* < 0.05.

### Supplementary Information

Supplementary Information.

## Data Availability

All generated data have been included in the published manuscript. For other information, contact with the corresponding author (Young-Hee Lim. yhlim@korea.ac.kr).
