# Supplementary material for: Surface-exposed chaperonin 60 derived from Propionibacterium freudenreichii MJ2 inhibits adipogenesis by decreasing the expression of C/EBPα/PPARγ
Source: Sci Rep. 2023 Nov 7;13:19251. doi: 10.1038/s41598-023-46436-x (PMC10630399; doi:10.1038/s41598-023-46436-x)
Supplement: Supplementary file 1 — Supplementary Information. [file 41598_2023_46436_MOESM1_ESM.docx]

**Supplementary information**

**Surface-exposed chaperonin 60 derived from *Propionibacterium freudenreichii* MJ2 inhibits adipogenesis by decreasing the expression of C/EBPα/PPARγ**

**Authors:**

**Mirae An^1^, Young-Hee Lim^1,2,3^**

**Affiliations:**

^1^Department of Healthcare Sciences, Graduate School, Korea University, Seoul, 02841, Republic of Korea

^2^School of Biosystems and Biomedical Sciences, Korea University, Seoul, 02841, Republic of Korea

^3^Department of Laboratory Medicine, Korea University Guro Hospital, Seoul, 08308, Republic of Korea

**Materials and methods**

**Ion exchange chromatography (IEX)**

The dialyzed crude surface proteins were separated by protein charge using fast proteins liquid chromatography (FPLC) (AKTA pure, GE Healthcare, IL, USA). The Q fast flow column (HiTrap^TM^ Q FF, 5 mL, GE Healthcare) was equilibrated with 20 mM Tris-HCl buffer (pH 8) before loading the surface proteins. The elution was performed using a rising gradient of NaCl (0‒0.8 M). The flow rate was 5 mL/min and the absorbance was measured at 280 nm using a spectrophotometer. After first ion-exchange chromatography, unbounded proteins were dialyzed with 10 mM Tris-HCl buffer (pH 8) overnight and reloaded to the Q fast flow column equilibrated with 10 mN Tris-HCl buffer (pH 8). The elution was performed by increasing the gradient of NaCl (0‒1 M). Each fraction was filtered with 0.45 μm pore filter (Minisart, Sartorius, Goettingen, Germany) and dialyzed with phosphate buffered saline (PBS) to remove NaCl before concentrating using ultracentrifugal filters (Amicon, MWCO 10 k, Sigma-Aldrich).

**Size exclusion chromatography (SEC)**

The proteins recovered from IEX were precipitated with trichloroacetic acid and resuspended with PBS. The proteins were separated by molecular size using FPLC. The proteins were subjected to a Superdex 75 Increase 10/35 column pre-equilibrated with PBS. The flow rate was 0.5 mL/min and analyzed by spectrophotometer at 280 nm. Each fraction was filtered with a 0.45 μm pore filter (Minisart) and concentrated using ultra centrifugal filters (Amicon, MWCO 10 k)

**Supplementary Table 1.** Extracted surface proteins from *P. freudenreichii* MJ2.

| **Accession** | **Protein description** | **Protein  score** | **MW  (kDa)** | **pI** | **#  Peptides** | **#  Unique  peptides** |
| --- | --- | --- | --- | --- | --- | --- |
| SCQ61269.1 | Large surface protein A | 1487 | 137 | 4.84 | 76 | 23 |
| SCQ60485.1 | Phosphopyruvate hydratase | 768 | 46 | 4.49 | 12 | 10 |
| WP_055345995.1 | Chaperonin 60 | 744 | 56 | 4.70 | 15 | 13 |
| SCQ67457.1 | Surface layer protein A (S-layer protein A) | 715 | 59 | 4.69 | 17 | 10 |
| SCQ60156.1 | Pyruvate carboxylase subunit B | 578 | 56 | 5.38 | 13 | 12 |
| SCQ60232.1 | 60 kDa chaperonin 1 | 576 | 57 | 4.72 | 15 | 9 |
| SCQ61251.1 | Succinate CoA transferase | 455 | 56 | 5.53 | 10 | 8 |
| SCQ59351.1 | Methylmalonyl-CoA mutase small subunit mutA | 452 | 70 | 5.03 | 11 | 9 |
| SCQ59862.1 | 10 kDa chaperonin | 446 | 11 | 4.95 | 12 | 8 |
| WP_055345899.1 | elongation factor Tu | 428 | 44 | 5.04 | 10 | 8 |
| SCQ59257.1 | Phospho acetyltransferase | 385 | 53 | 4.99 | 7 | 7 |
| SCQ62383.1 | Hsp20/alpha crystallin family protein | 380 | 17 | 4.93 | 10 | 9 |
| SCQ61146.1 | Succinate dehydrogenase flavoprotein subunit | 377 | 75 | 6.00 | 7 | 7 |
| SCQ67187.1 | ATP-dependent chaperone protein ClpB | 331 | 94 | 5.06 | 7 | 7 |
| SCQ60211.1 | 30S ribosomal protein S7 | 315 | 17 | 10.34 | 7 | 7 |
| SCQ60593.1 | Chaperone protein DnaK | 308 | 67 | 4.66 | 7 | 7 |
| SCQ61734.1 | 2,5-diketo-D-gluconic acid reductase A | 287 | 31 | 5.02 | 6 | 6 |
| SCQ59689.1 | Malate dehydrogenase | 275 | 35 | 4.95 | 5 | 4 |
| SCQ61014.1 | Translation elongation factor Ts | 272 | 29 | 4.79 | 4 | 4 |
| SCQ60207.1 | Translation elongation factor G | 260 | 77 | 4.83 | 6 | 6 |
| SCQ60942.1 | Glyceraldehyde 3-phosphate dehydrogenase gap | 260 | 38 | 5.46 | 4 | 4 |
| SCQ66419.1 | Triosephosphate isomerase 1 | 224 | 28 | 5.05 | 5 | 4 |
| pdb\|1ON3\|A | Chain A, Methylmalonyl-CoA carboxyltransferase 12S subunit | 223 | 57 | 5.38 | 4 | 4 |
| SCQ60201.1 | Iron-sulfur cluster-binding protein | 204 | 58 | 6.75 | 6 | 4 |
| SCQ60108.1 | 30S ribosomal protein S5 | 200 | 22 | 10.27 | 7 | 3 |
| SCQ59355.1 | Methylmalonyl-CoA mutase large subunit mutB | 199 | 80 | 5.29 | 5 | 4 |
| SCQ60031.1 | 30S ribosomal protein S4 | 192 | 23 | 10.13 | 4 | 3 |
| SCQ59933.1 | Inositol 1-phosphate synthase | 187 | 40 | 4.77 | 4 | 3 |
| SCQ69359.1 | Fructose-bisphosphate aldolase class 1 | 168 | 32 | 5.02 | 4 | 4 |
| SCQ60370.1 | NADH dehydrogenase subunit E | 167 | 26 | 4.87 | 3 | 2 |
| SCQ61150.1 | Succinate dehydrogenase/fumarate reductase iron-sulfur subunit | 167 | 28 | 5.61 | 4 | 3 |
| SCQ61012.1 | 30S ribosomal protein S2 | 165 | 36 | 5.12 | 3 | 3 |
| SCQ61320.1 | Acetate kinase | 163 | 43 | 5.44 | 4 | 4 |
| SCQ61805.1 | Pyruvate-flavodoxin oxidoreductase (Precursor) | 163 | 138 | 5.19 | 5 | 4 |
| SCQ60169.1 | 2,3-bisphosphoglycerate-dependent phosphoglycerate mutase | 160 | 28 | 5.78 | 3 | 3 |
| SCQ60098.1 | Fructose-bisphosphate aldolase, class II | 156 | 37 | 5.21 | 3 | 3 |
| SCQ60115.1 | 50S ribosomal protein L6 | 155 | 20 | 9.42 | 3 | 3 |
| SCQ76885.1 | Cell division protein FtsZ | 153 | 43 | 4.49 | 4 | 3 |
| SCQ62637.1 | 30S ribosomal protein S18 | 150 | 9 | 11.05 | 3 | 3 |
| SCQ60901.1 | 30S ribosomal protein S1 | 149 | 54 | 4.61 | 2 | 2 |
| WP_055345897.1 | 50S ribosomal protein L24 | 148 | 13 | 10.36 | 3 | 3 |
| SCQ62302.1 | Cold shock-like protein CspA | 139 | 7 | 4.82 | 3 | 3 |
| SCQ61299.1 | Peptidylprolyl isomerase | 136 | 36 | 4.98 | 3 | 3 |

Supplementary Table 1. Continued.

| **Accession** | **Protein description** | **Protein  score** | **MW  (kDa)** | **pI** | **#  Peptides** | **#  Unique  peptides** |
| --- | --- | --- | --- | --- | --- | --- |
| SCQ60699.1 | Fumarate hydratase class II | 133 | 51 | 5.34 | 3 | 3 |
| SCQ59685.1 | Isocitrate dehydrogenase [NADP] | 133 | 45 | 5.20 | 2 | 2 |
| SCQ62863.1 | Catalase | 133 | 54 | 5.01 | 3 | 3 |
| SCQ59955.1 | 30S ribosomal protein S9 | 133 | 19 | 9.93 | 3 | 3 |
| SCQ60023.1 | 50S ribosomal protein L17 | 132 | 21 | 9.58 | 3 | 3 |
| SCQ60215.1 | 30S ribosomal protein S12 | 132 | 14 | 11.49 | 3 | 3 |
| SCQ61798.1 | Dihydroorotate dehydrogenase 2 | 131 | 40 | 4.80 | 4 | 3 |
| SCQ60142.1 | 50S ribosomal protein L29 | 130 | 9 | 6.59 | 3 | 2 |
| SCQ60027.1 | DNA-directed RNA polymerase, alpha subunit | 123 | 37 | 4.35 | 2 | 2 |
| SCQ61208.1 | 50S ribosomal protein L35 | 119 | 7 | 11.77 | 4 | 3 |
| SCQ60118.1 | 30S ribosomal protein S8 | 118 | 15 | 9.33 | 3 | 3 |
| SCQ62128.1 | ATP synthase subunit beta | 115 | 53 | 4.67 | 3 | 3 |
| SCQ59166.1 | Trigger factor | 113 | 57 | 4.61 | 4 | 2 |
| SCQ60155.1 | 50S ribosomal protein L22 | 107 | 17 | 10.52 | 4 | 3 |
| SCQ60464.1 | Transaldolase | 106 | 38 | 4.84 | 2 | 2 |
| SCQ60099.1 | 50S ribosomal protein L15 | 105 | 15 | 10.32 | 2 | 2 |
| SCQ59150.1 | ATP-dependent Clp protease proteolytic subunit 1 | 101 | 25 | 4.72 | 3 | 3 |
| SCQ60180.1 | 30S ribosomal protein S10 | 98 | 12 | 9.60 | 4 | 3 |
| SCQ60133.1 | 50S ribosomal protein L14 | 90 | 13 | 10.01 | 2 | 2 |
| SCQ60374.1 | NADH-quinone oxidoreductase subunit D | 83 | 51 | 4.81 | 1 | 1 |
| SCQ60589.1 | Protein GrpE | 82 | 23 | 4.69 | 3 | 3 |
| SCQ61688.1 | Phosphofructokinase (Precursor) | 80 | 43 | 4.89 | 2 | 2 |
| SCQ60252.1 | 50S ribosomal protein L11 | 78 | 15 | 9.49 | 1 | 1 |
| SCQ59960.1 | 50S ribosomal protein L13 | 76 | 16 | 9.65 | 2 | 2 |
| SCQ61447.1 | Vitamin B12-dependent ribonucleotide reductase | 75 | 106 | 4.89 | 2 | 2 |
| SCQ62315.1 | Tryptophanyl-tRNA synthetase II | 74 | 43 | 5.78 | 2 | 2 |
| SCQ61445.1 | UDP-glucose 4-epimerase, galE | 73 | 35 | 5.53 | 2 | 2 |
| SCQ60040.1 | 30S ribosomal protein S13 | 73 | 14 | 10.73 | 1 | 1 |
| SCQ60235.1 | 50S ribosomal protein L10 | 72 | 22 | 4.52 | 2 | 1 |
| SCQ62466.1 | Glycerophosphodiester phosphodiesterase family protein | 72 | 30 | 4.67 | 2 | 2 |
| SCQ60111.1 | 50S ribosomal protein L18 | 71 | 14 | 11.40 | 1 | 1 |
| SCQ60036.1 | 30S ribosomal protein S11 | 71 | 14 | 11.54 | 1 | 1 |
| SCQ60009.1 | Superoxide dismutase | 71 | 23 | 5.28 | 2 | 2 |
| SCQ59076.1 | 50S ribosomal protein L27 | 67 | 9 | 11.65 | 2 | 2 |
| SCQ60289.1 | Alkyl hydroperoxide reductase | 65 | 21 | 4.62 | 1 | 1 |
| SCQ62164.1 | 50S ribosomal protein L31 | 64 | 8 | 9.24 | 1 | 1 |
| SCQ60172.1 | 50S ribosomal protein L4 | 63 | 25 | 9.78 | 1 | 1 |
| SCQ60469.1 | Bifunctional protein GlmU | 62 | 55 | 5.11 | 1 | 1 |
| SCQ60363.1 | NADH dehydrogenase, G subunit | 61 | 86 | 5.11 | 1 | 1 |
| SCQ62408.1 | Acyl carrier protein | 57 | 9 | 4.07 | 2 | 2 |
| SCQ66762.1 | Glutamate--ammonia ligase | 57 | 54 | 4.84 | 2 | 1 |
| SCQ61064.1 | 30S ribosomal protein S15 | 56 | 10 | 9.91 | 2 | 2 |
| SCQ61042.1 | Transcription termination factor NusA | 55 | 38 | 5.05 | 2 | 2 |

Supplementary Table 1. Continued.

| **Accession** | **Protein description** | **Protein  score** | **MW  (kDa)** | **pI** | **#  Peptides** | **#  Unique  peptides** |
| --- | --- | --- | --- | --- | --- | --- |
| SCQ62408.1 | Acyl carrier protein | 57 | 9 | 4.07 | 2 | 2 |
| SCQ66762.1 | Glutamate--ammonia ligase | 57 | 54 | 4.84 | 2 | 1 |
| SCQ61064.1 | 30S ribosomal protein S15 | 56 | 10 | 9.91 | 2 | 2 |
| SCQ61042.1 | Transcription termination factor NusA | 55 | 38 | 5.05 | 2 | 2 |
| SCQ62396.1 | Pyruvate dehydrogenase E1 component | 53 | 102 | 5.46 | 1 | 1 |
| SCQ58995.1 | Chaperone protein DnaJ 2 | 53 | 43 | 6.17 | 1 | 1 |
| SCQ60122.1 | 30S ribosomal protein S14 type Z | 51 | 7 | 11.29 | 1 | 1 |
| SCQ60176.1 | 50S ribosomal protein L3 | 50 | 23 | 10.19 | 2 | 1 |
| SCQ60719.1 | FAD linked oxidase protein | 49 | 102 | 5.68 | 1 | 1 |
| SCQ60380.1 | NADH-quinone oxidoreductase subunit C | 48 | 29 | 5.34 | 1 | 1 |
| SCQ61879.1 | Aminopeptidase C | 48 | 50 | 5.06 | 1 | 1 |
| SCQ60801.1 | Phosphoribosylformylglycinamidine synthase subunit PurS | 48 | 9 | 5.09 | 1 | 1 |
| SCQ60049.1 | Translation initiation factor IF-1 | 46 | 8 | 9.52 | 1 | 1 |
| SCQ60355.1 | NADH-quinone oxidoreductase subunit I | 46 | 22 | 5.70 | 1 | 1 |
| SCQ60471.1 | Ribose-phosphate diphosphokinase | 45 | 37 | 6.07 | 1 | 1 |
| SCQ60126.1 | 50S ribosomal protein L5 | 45 | 25 | 9.57 | 1 | 1 |
| SCQ60319.1 | Type I antifreeze protein | 43 | 13 | 4.86 | 1 | 1 |
| SCQ60889.1 | Pyruvate kinase | 43 | 52 | 5.01 | 1 | 1 |
| SCQ59217.1 | DTDP-glucose 4,6-dehydratase RmlB | 43 | 37 | 5.45 | 1 | 1 |
| SCQ59853.1 | Inosine-5~-monophosphate dehydrogenase GuaB | 41 | 54 | 5.49 | 1 | 1 |
| SCQ60153.1 | Glutamate-1-semialdehyde 2,1-aminomutase hemL | 40 | 46 | 5.10 | 1 | 1 |
| SCQ60816.1 | Transcriptional regulator, AsnC family | 39 | 10 | 4.37 | 1 | 1 |
| SCQ61274.1 | UDP-galactopyranose mutase glf | 39 | 45 | 5.29 | 1 | 1 |
| SCQ61198.1 | Phosphoribosyl-AMP pyrophosphatase hisE | 39 | 10 | 4.68 | 1 | 1 |
| SCQ60787.1 | Phage shock protein A, PspA | 38 | 29 | 5.26 | 1 | 1 |
| SCQ63796.1 | Ribonuclease P protein component | 38 | 15 | 12.09 | 1 | 1 |
| SCQ61153.1 | 50S ribosomal protein L19 | 36 | 13 | 10.36 | 1 | 1 |
| SCQ62125.1 | ATP synthase F1, epsilon subunit | 35 | 17 | 4.61 | 1 | 1 |
| SCQ59369.1 | Dihydrofolate reductase | 34 | 21 | 5.00 | 1 | 1 |
| WP_048768990.1 | phosphoglucosamine mutase | 34 | 47 | 4.91 | 1 | 1 |
| SCQ60167.1 | 50S ribosomal protein L23 | 33 | 12 | 9.64 | 1 | 1 |
| SCQ60257.1 | Transcription antitermination protein nusG | 31 | 32 | 4.28 | 1 | 1 |
| SCQ60281.1 | 50S ribosomal protein L33 | 31 | 7 | 10.44 | 1 | 1 |
| SCQ60013.1 | Resuscitation-promoting factor RpfB | 30 | 38 | 5.27 | 1 | 1 |
| SCQ59630.1 | NAD(P)() transhydrogenase (AB-specific), alpha subunit | 30 | 54 | 5.39 | 1 | 1 |
| SCQ62375.1 | 4-aminobutyrate aminotransferase gabT | 29 | 47 | 5.29 | 1 | 1 |
| SCQ61459.1 | Anaerobic glycerol-3-phosphate dehydrogenase subunit C | 29 | 52 | 7.81 | 1 | 1 |
| SCQ62439.1 | Inositol 2-dehydrogenase 2 | 29 | 37 | 5.04 | 1 | 1 |
| SCQ59648.1 | Methionyl-tRNA synthetase (Methionine--tRNA ligase) (MetRS) | 29 | 72 | 4.95 | 1 | 1 |
| SCQ59239.1 | Dihydroxyacetone kinase, DhaK subunit | 28 | 35 | 4.66 | 1 | 1 |
| SCQ61343.1 | Ketol-acid reductoisomerase 2 | 28 | 37 | 5.01 | 1 | 1 |
| SCQ60944.1 | Phosphoglycerate kinase | 27 | 43 | 5.21 | 1 | 1 |
| SCQ66117.1 | Signal recognition particle receptor FtsY | 26 | 40 | 4.78 | 1 | 1 |

Supplementary Table 1. Continued.

| **Accession** | **Protein description** | **Protein  score** | **MW  (kDa)** | **pI** | **#  Peptides** | **#  Unique  peptides** |
| --- | --- | --- | --- | --- | --- | --- |
| SCQ60539.1 | Amidophosphoribosyltransferase | 25 | 56 | 5.49 | 1 | 1 |
| SCQ67267.1 | Hydrolase, TatD family | 25 | 37 | 5.68 | 1 | 1 |
| SCQ61205.1 | Translation initiation factor IF-3 | 24 | 28 | 9.45 | 1 | 1 |
| SCQ62094.1 | Phosphoglucomutase PgmA | 24 | 59 | 5.03 | 1 | 1 |
| SCQ61221.1 | Phenylalanine--tRNA ligase beta subunit | 23 | 91 | 4.75 | 1 | 1 |
| SCQ59102.1 | DNA polymerase III, beta subunit | 22 | 42 | 4.80 | 1 | 1 |

**Supplementary Table 2.** Functional pathway enrichments of the network of surface proteins in KEGG.

| **Pathway ID** | **Description** | **Strength** | **Gene symbol** |
| --- | --- | --- | --- |
| pfr03010 | Ribosome | 1.07 | rpmG, rplK, rplA, rplJ, rpsL, rpsG, rpsJ, rplC, rplD, rplW, rplV, rpmC, rplN, rplX, rplE, rpsN1, rpsH, rplR, rpsE, rplO, rpsM, rpsK, rpsD, rplQ, rplM, rspI, rpmA, rpmE, rpmI, rplS, rpsO, rpsB, rpsA, rpsR |
| pfr00521 | Streptomycin biosynthesis | 1.01 | rmlB, pgm1, iolG1, CBL57481.1 |
| pfr00680 | Methane metabolism | 0.92 | ackA, pta, mdh, eno1, gpm2, fba1, fba2 |
| pfr00010 | Glycolysis / Gluconeogenesis | 0.88 | nifJ2, aceE, pgm1, lpd, tpi1, pgk, gap, pyk1, eno1, gpm2, fba1, fba2 |
| pfr00640 | Propanoate metabolism | 0.77 | ackA, pta, mutB, mutA, gabT, lpd, mmdA, CBL57379.1 |
| pfr00020 | Citrate cycle (TCA cycle) | 0.76 | mdh, icd, nifJ2, aceE, lpd, sdhB3, sdhA3, fumC |
| pfr03018 | RNA degradation | 0.74 | dnaK2, groL1, eno1, groL2 |
| pfr00280 | Valine, leucine and isoleucine degradation | 0.74 | mutB, mutA, gabT, lpd |
| pfr00620 | Pyruvate metabolism | 0.71 | ackA, pta, mdh, nifJ2, aceE, lpd, pyk1, fumC |
| pfr01200 | Carbon metabolism | 0.7 | ackA, pta, tal1, mdh, icd, nifJ2, mutB, mutA, aceE, lpd, sdhB3, sdhA3, tpi1, pgk, gap, pyk1, fumC, eno1, prs, gpm2, fba1, katA, fba2 |
| pfr00190 | Oxidative phosphorylation | 0.67 | nuoC, nuoD, nuoE, nuoG, nuoI, atpH, atpD, sdhB3, sdhA3 |
| pfr00630 | Glyoxylate and dicarboxylate metabolism | 0.65 | mdh, mutB, mutA, lpd, glnA, katA |
| pfr00520 | Amino sugar and nucleotide sugar metabolism | 0.61 | glf, galE1, glmM, pgm1, glmU |
| pfr00030 | Pentose phosphate pathway | 0.57 | tal1, pgm1, prs, fba1, fba2 |
| pfr01120 | Microbial metabolism in diverse environments | 0.55 | ackA, pta, tal1, mdh, icd, nifJ2, mutB, mutA, aceE, gabT, pgm1, lpd, sdhB3, sdhA3, tpi1, pgk, gap, pyk1, glnA, dapD, fumC, eno1, prs, gpm2, hemL1, iolG1, fba1, katA, fba2 |
| pfr01130 | Biosynthesis of antibiotics | 0.48 | rmlB, purS, purF, tal1, glmM, mdh, icd, nifJ2, aceE, pgm1, lpd, ilvC, sdhB3, sdhA3, tpi1, pgk, gap, pyk1, fumC, eno1, prs, glmU, gpm2, iolG1, fba1, CBL57481.1, katA, fba2 |
| pfr00230 | Purine metabolism | 0.48 | dnaN, purS, purF, rpoA, guaB1, pgm1, nrdJ, pyk1, prs |
| pfr01230 | Biosynthesis of amino acids | 0.42 | tal1, icd, ilvC, hisE, tpi1, pgk, gap, pyk1, glnA, dapD, eno1, prs, gpm2, fba1, fba2 |
| pfr01110 | Biosynthesis of secondary metabolites | 0.35 | purS, purF, tal1, guaB1, mdh, icd, aceE, pgm1, lpd, glpC, ilvC, hisE, sdhB3, sdhA3, tpi1, pgk, gap, pyk1, fumC, eno1, prs, gpm2, hemL1, fba1, katA, fba2 |
| pfr01100 | Metabolic pathways | 0.32 | dnaN, ackA, pta, galE1, purS, purF, tal1, nuoC, nuoD, nuoE, nuoG, nuoI, rpoA, glmM, guaB1, mdh, icd, nifJ2, folA, mutB, mutA, dhaK, aceE, gabT, atpH, atpD, pgm1, lpd, pyrD, nrdJ, ilvC, hisE, sdhB3, sdhA3, tpi1, pgk, gap, pyk1, glnA, dapD, fumC, glpD2, eno1, prs, glmU, gpm2, hemL1, iolG1, fba1, CBL57481.1, pntB, fba2 |

**Supplementary Table 3.** Annotated keywords of surface proteins in GO database (UniProt).

| **Keyword ID** | **Description** | **Strength** | **Gene symbol** |
| --- | --- | --- | --- |
| KW-0816 | Tricarboxylic acid cycle | 1.13 | mdh, icd, fumC |
| KW-0689 | Ribosomal protein | 1.07 | rpmG, rplK, rplA, rplJ, rpsL, rpsG, rpsJ, rplC, rplD, rplW, rplV, rpmC, rplN, rplX, rplE, rpsN1, rpsH, rplR, rpsE, rplO, rpsM, rpsK, rpsD, rplQ, rplM, rspI, rpmA, rpmE, rpmI, rplS, rpsO, rpsB, rpsA, rpsR |
| KW-0699 | rRNA-binding | 1.04 | rplK, rplA, rplJ, rpsL, rpsG, rplC, rplD, rplW, rplV, rplN, rplX, rplE, rpsN1, rpsH, rplR, rpsE, rplO, infA, rpsM, rpsK, rpsD, rpmE, rpsO, rpsR |
| KW-0251 | Elongation factor | 1.03 | fusA, tuf, tsf |
| KW-0820 | tRNA-binding | 0.95 | rplA, rpsL, rpsG, rplE, rpsM, pheT |
| KW-0324 | Glycolysis | 0.95 | tpi1, pgk, pyk1, eno1, gpm2 |
| KW-0143 | Chaperone | 0.92 | dnaK2, grpE2, dnaJ2, groS, groL1, tig, clpB_2, groL2 |
| KW-0694 | RNA-binding | 0.89 | rplK, rplA, rplJ, rpsL, rpsG, rplC, rplD, rplW, rplV, rplN, rplX, rplE, rpsN1, rpsH, rplR, rpsE, rplO, infA, rpsM, rpsK, rpsD, rpmE, pheT, rpsO, nusA, rpsR, rnpA |
| KW-1278 | Translocase | 0.88 | nuoC, nuoD, nuoG, nuoI, atpD, pntB |
| KW-0346 | Stress response | 0.83 | dnaK2, grpE2, dnaJ2, groS, groL1, hsp20_2, clpB_2, groL2 |
| KW-0874 | Quinone | 0.77 | nuoC, nuoD, nuoG, nuoI |
| KW-0520 | NAD | 0.67 | galE1, nuoC, nuoD, nuoG, nuoI, guaB1, mdh, lpd, gap, pntB |
| KW-0648 | Protein biosynthesis | 0.54 | fusA, tuf, infA, metG, trpS, pheT, infC, tsf |
| KW-0413 | Isomerase | 0.53 | glf, galE1, glmM, mutB, mutA, tig, pgm1, ilvC, CBL56873.1, tpi1, gpm2, hemL1, CBL57481.1 |
| KW-0408 | Iron | 0.53 | purF, nuoE, nuoG, nuoI, nifJ2, PF47, katA |
| KW-0560 | Oxidoreductase | 0.46 | dkgA, nuoC, nuoD, nuoE, nuoG, nuoI, ahpC, sodA, guaB1, mdh, icd, nifJ2, folA, aceE, lpd, pyrD, glpC, nrdJ, ilvC, sdhB3, sdhA3, gap, iolG1, pntB, katA |
| KW-0963 | Cytoplasm | 0.45 | dnaN, ackA, purS, grpE2, dnaJ2, tal1, ahpC, fusA, tuf, infA, groS, groL1, tig, clpP1, acpP, cspA, pheT, infC, ftsY, nusA, tsf, tpi1, pgk, ftsZ, dapD, fumC, eno1, prs, glmU, clpB_2, groL2, hemL1 |
| KW-0460 | Magnesium | 0.45 | ackA, purF, glmM, icd, aceE, pgm1, ilvC, pheT, pyk1, dapD, eno1, prs, glmU |
| KW-0479 | Metal-binding | 0.4 | ackA, dnaJ2, purF, nuoE, nuoG, nuoI, rpsN1, sodA, glmM, guaB1, icd, nifJ2, aceE, rpmE, pgm1, ilvC, pheT, PF47, dapD, eno1, prs, glmU, fba1, katA |
| KW-0175 | Coiled coil | 0.33 | dnaK2, grpE2, nuoD, rplQ, tig, nrdJ, infC, ftsY, tsf, rpsB, rpsA, ftsZ, pspA, clpB_2, groL2, CBL57379.1 |


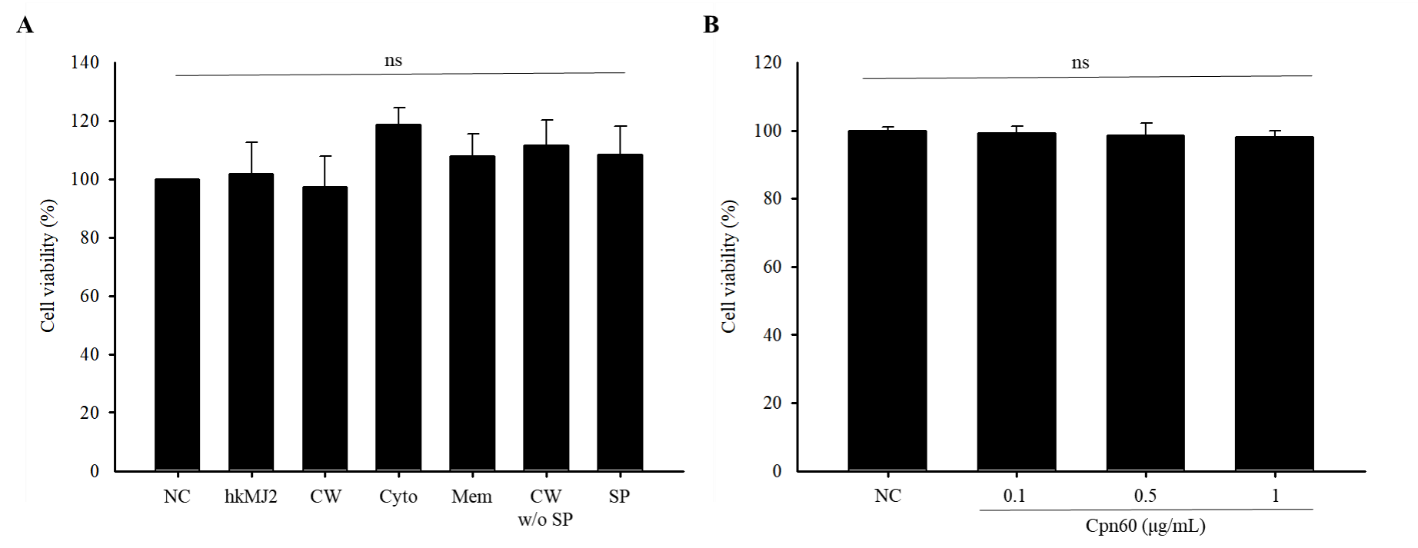


**Supplementary Fig. 1.** Effects of various components from *P. freudenreichii* MJ2 on 3T3-L1 cells viability compared with the negative control (NC). 3T3-L1 cells were treated with 1 × 10^8^ cells/mL of heat-killed *P. freudenreichii* MJ2 (hkMJ2) and various cell fractions from *P. freudenreichii* MJ2: cell wall (CW), cytoplasm (Cyto), membrane (Mem), cell wall without surface proteins (CW w/o SP), and surface proteins (SP), at the concentrations originated in the same cells count with hkMJ2 (A) and 3T3-L1 cells were treated with various concentrations of Cpn60 (B). NC, negative control; ns, no significance.


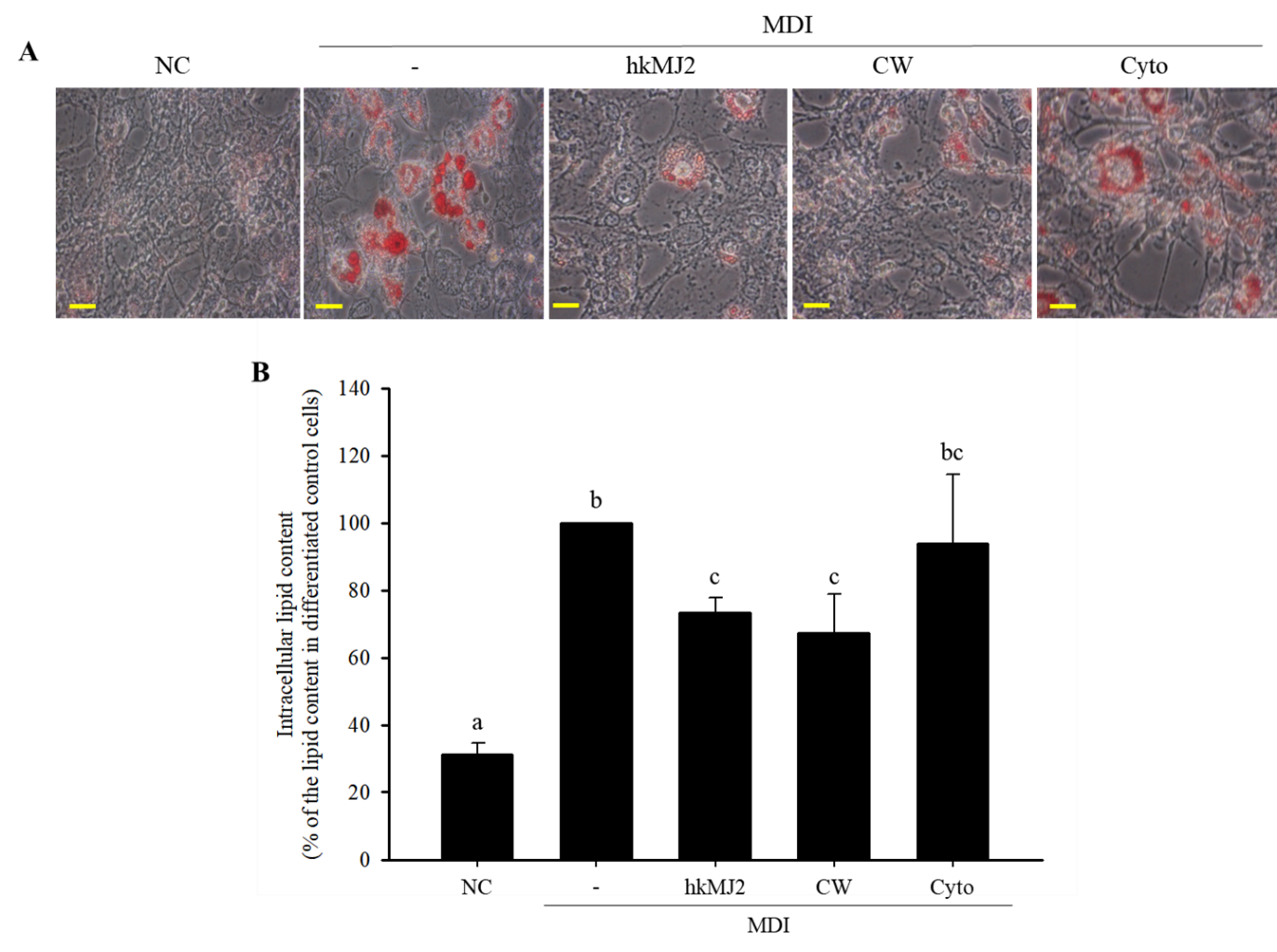


**Supplementary Fig. 2.** Inhibitory effects of cell fractions from *P. freudenreichii* MJ2 on lipid accumulation in 3T3-L1 cells. 3T3-L1 cells were treated with 1 × 10^8^ cells/mL of heat-killed *P. freudenreichii* MJ2 (hkMJ2) and cell wall (CW) and cytoplasm (Cyto) fractions from *P. freudenreichii* MJ2 at the concentrations originated in the same cells count with hkMJ2. Lipid droplets of the differentiated cells were visualized by oil red o staining (400× magnification) and the scale bars indicate 0.5 mm (A) and quantified (B). The *p* values are determined by ANOVA and Tukey’s honest significant difference (HSD) test and different letters indicate statistically significant differences (*p* < 0.05). NC, negative control; MDI, 1 μM dexamethasone, 0.5 mM IBMX, and 5 μg/mL insulin.


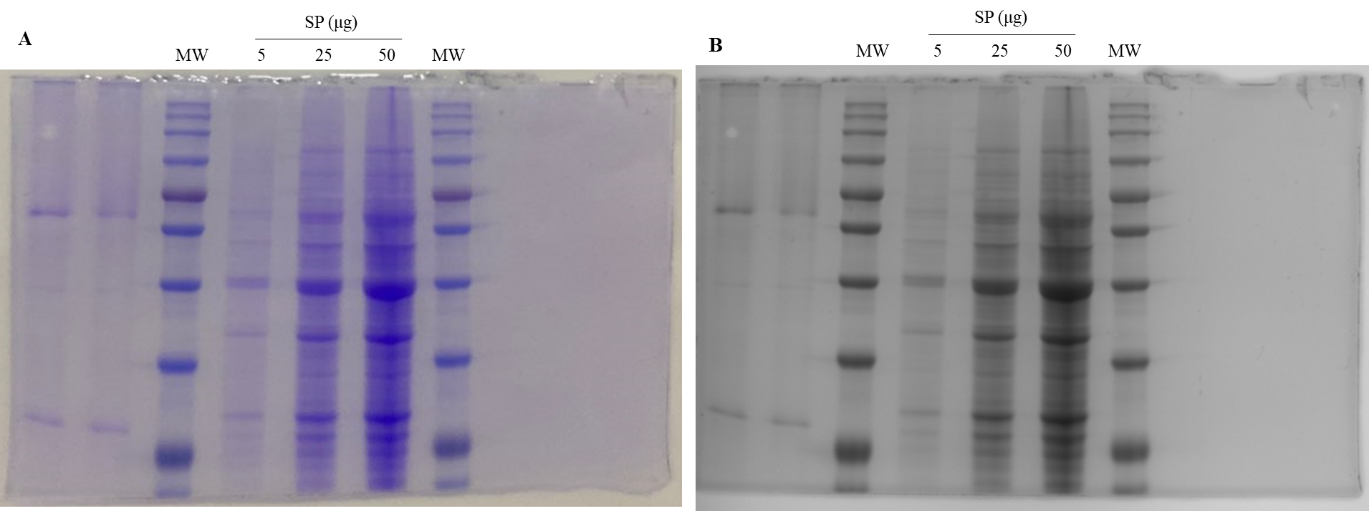


**Supplementary Fig. 3.** The full-length images of the SP proteins shown in Fig. 2A.


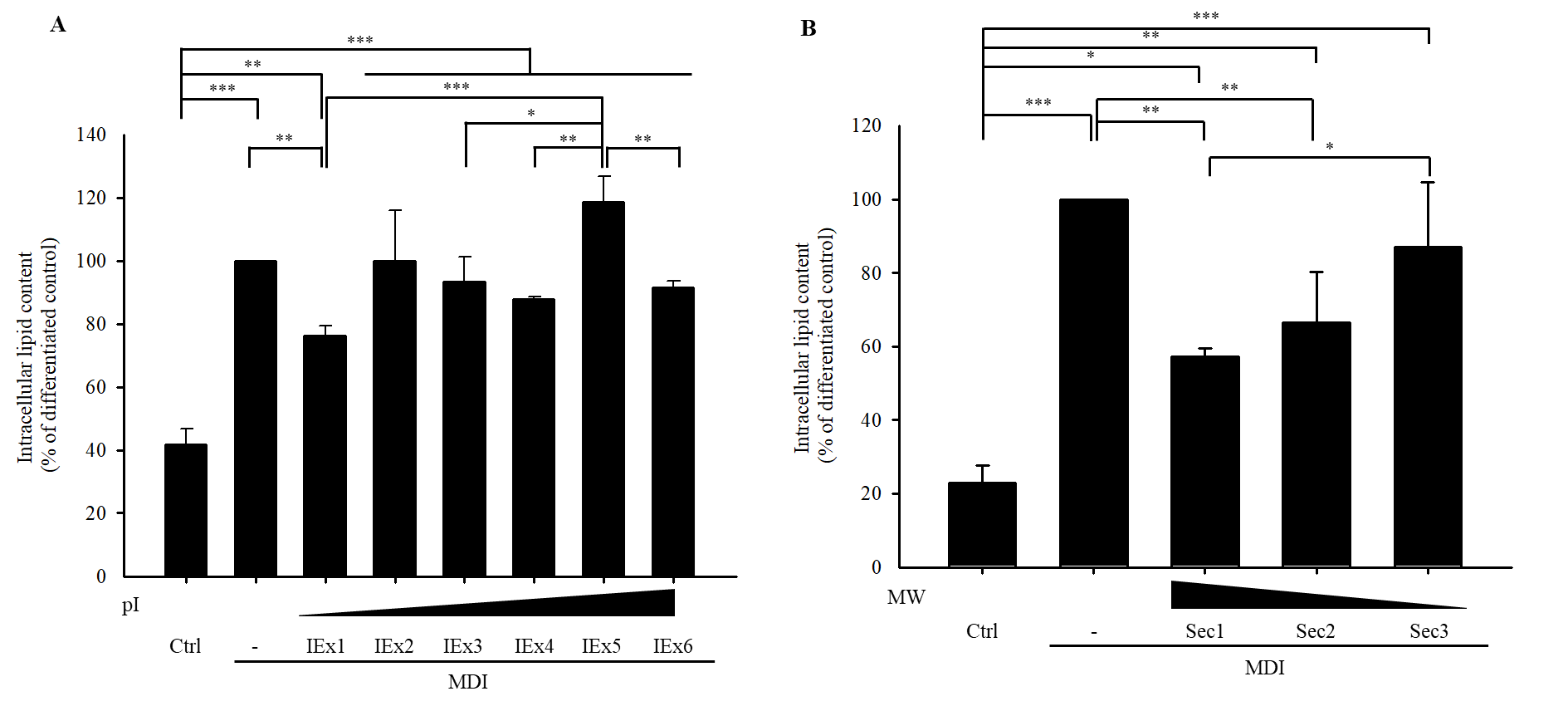


**Supplementary Fig. 4.** Inhibitory effect of the fractions of surface proteins (SP) *from P. freudenreichii* MJ2 separated using ion-exchange chromatography and size exclusive chromatography on lipid accumulation in 3T3-L1 adipocytes. 3T3-L1 preadipocytes were differentiated by treatment with differentiation-inducing medium containing MDI (IBMX + dexamethasone + insulin), and the fractions of surface proteins. Lipid accumulation was quantified by measuring lipid droplets stained with oil red O and comparing them with the cells treated with MDI alone. Lipid accumulations of the cells treated with the fractions separated by ion exchange chromatography (A) and size exclusive chromatography (B). The data indicate the mean ± SD of three independent experiments. Expected binding pI range: IEx1, 3.5‒5; IEx2, 4‒5; IEx3, 5‒6.5; IEx4, 6‒7; IEx5, 6.5‒8; IEx6, 8 <. Expected protein size: Sec1, 35 kDa <; Sec2, 35 kDa >; Sec3, 20 kDa >. The *p* values are determined by ANOVA and Tukey’s HSD test and differences were considered significant at **p* < 0.05, ***p* < 0.01, and ****p* < 0.001.


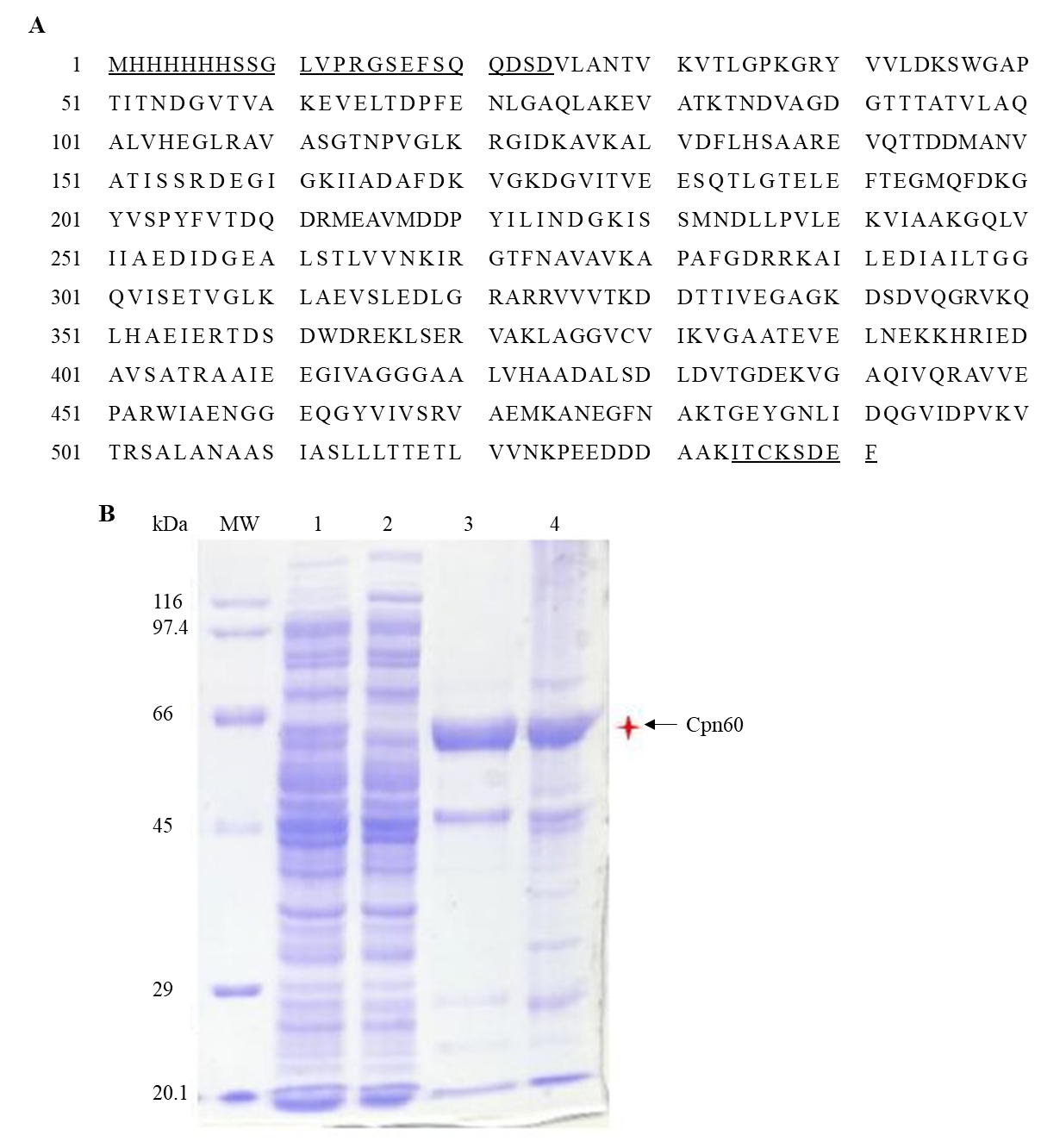


**Supplementary Fig. 5.** Sequences and purification of the recombinant chaperonin 60 (Cpn60). Chaperonin 60 was expressed by transformed into *E. coli* and purified using an automated protein production system. (A) Amino acid sequences of chaperonin Cpn60 are capitalized and vector sequences are underlined. (B) Results of SDS-PAGE. Lane 1, whole proteins, lane 2: the proteins except purified chaperonin 60, lane 3: the purified chaperonin 60, lane 4: bead (resuspension), MW: molecular marker.


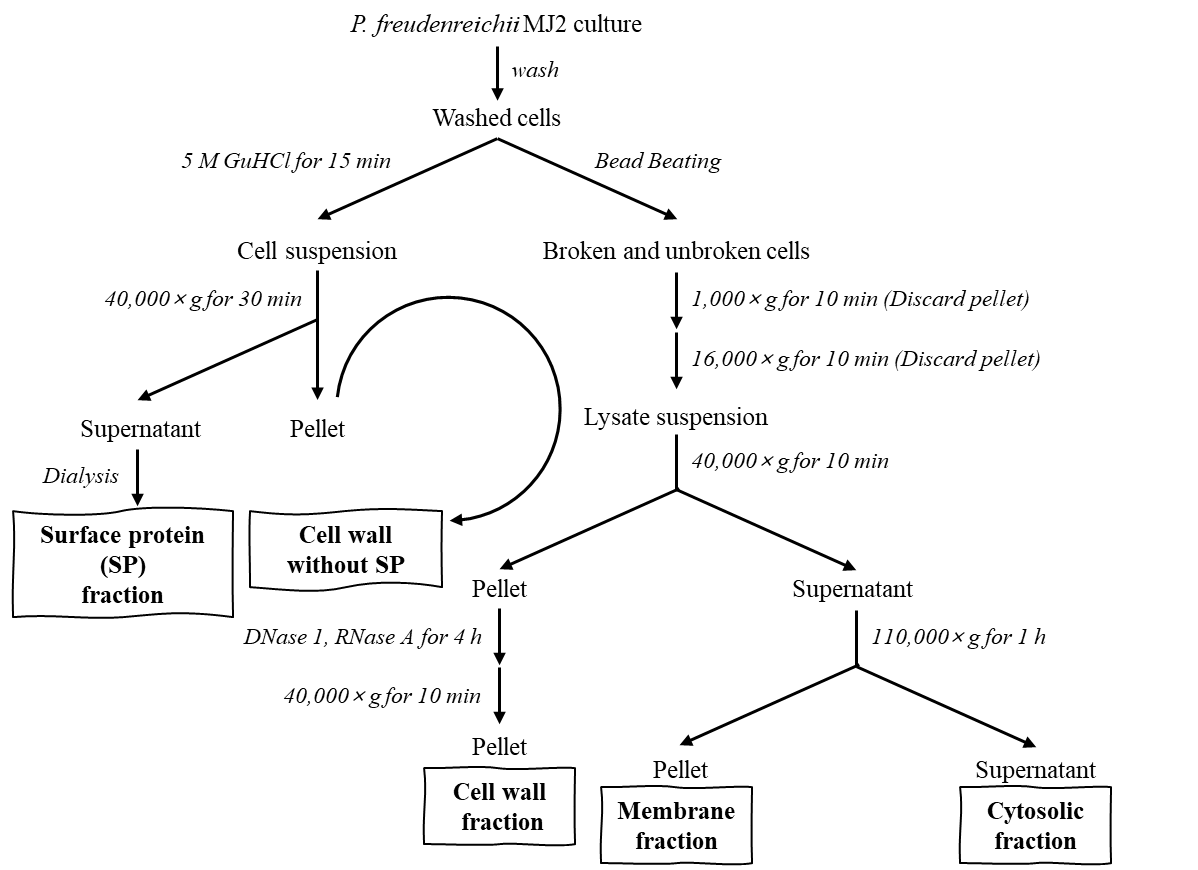


**Supplementary Fig. 6.** The scheme for preparation of cell fractions from *P. freudenreichii* MJ2.

**
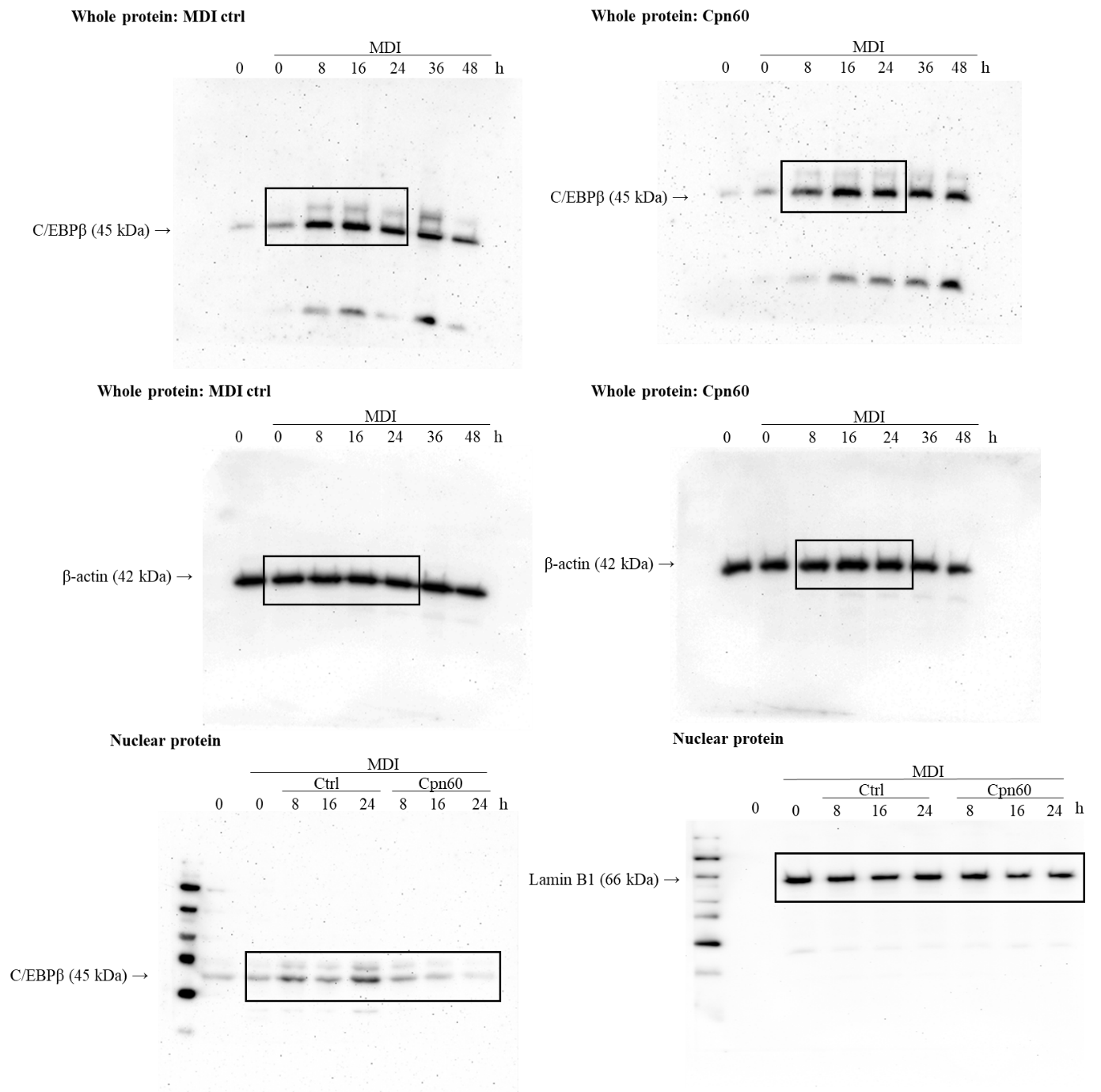
**

**Supplementary Fig. 7.** The full-length blots of the proteins shown in Fig. 7. The boxes are the bands shown in Fig. 7.
